# Status, and pathways to implementing the revitalised PHC approach in the WHO African Region: a mixed-methods study

**DOI:** 10.1136/bmjph-2026-005396

**Published:** 2026-08-07

**Authors:** Humphrey Cyprian Karamagi, Sokona Sy, Joseph Mung’atu, Anabay Mamo, Hillary Kipruto, Isabella Maina, Benson Droti

**Affiliations:** 1 World Health Organization Regional Office for Africa, Brazzaville, Congo; 2 Jomo Kenyatta University of Agriculture and Technology, Nairobi, Kenya

**Keywords:** Public Health Practice, Statistics as Topic, Health Services Accessibility

## Abstract

**Background:**

The revitalised primary healthcare (PHC) approach aims to enhance attainment of health results. However, implementation challenges persist, due among other issues, to different ways its different drivers are interpreted and applied in practice. In this study, we explore the status, and most appropriate ways the PHC approach drivers need to be applied. Through this, we propose country-specific priorities and optimal pathways for its implementation.

**Methods:**

We develop a measure of the status of its different drivers—3 components, 4 strategic and 10 operational levers—in each country of the WHO African Region, using perspectives from stakeholders in each country. We use this information to derive specific indices for each driver, which are then used to construct a relative measure of PHC implementation. We apply structural equation modelling to analyse the optimal pathway these different drivers use to best impact the health results chain.

**Results:**

On a scale of 0–1, representing no—full relative implementation of the revitalised PHC approach drivers, the scores of the countries in the region range from 0.37 to 0.90. Similarly, the scores for the constituent PHC drivers ranged from 0.71 for the PHC components average, down to 0.60 for the operational levers. The optimal pathway to achieving health results is one where the PHC components influence the status of the strategic levers, which influence health investments either by focusing on the operational levers specifically or the functionality of the system to achieve desired results.

**Conclusions:**

The study shows that the adoption of the revitalised PHC approach should go beyond policy rhetoric and focus on integrating and sequencing its drivers and constructs into targeted areas of the results chain for maximal impact on health results.

**WHAT IS ALREADY KNOWN ON THIS TOPIC**

- The countries in Africa have taken up the revitalised primary healthcare (PHC) approach as a key part of how they will attain their health results. However, decision-makers grapple with its integration into health systems to positively impact their desired health results.

**WHAT THIS STUDY ADDS**

- To our knowledge, this study provides the first region-wide empirical assessment of how countries in the WHO African Region are implementing the revitalised PHC approach and identifies the optimal pathways through which PHC drivers influence health results.

**HOW THIS STUDY MIGHT AFFECT RESEARCH, PRACTICE OR POLICY**

- Countries can focus on the revitalised PHC drivers that contribute most effectively in their setting. This study contributes in closing that gap by guiding countries in areas where most focus is needed to speed up progress.

## Introduction

 Since the Alma-Ata Declaration of 1978, primary healthcare (PHC) has remained central to global health development, providing a framework for delivering fair and effective health interventions.^1^ However, its implementation over time faced challenges such as political resistance, resource constraints and the growing focus on specific high-impact interventions, among others.^2,3^

Over time, these challenges have led to it being interpreted differently. During the 1980s, the focus was on a selective PHC approach that focused on high-impact interventions.^2^ By the 1990s, economic challenges in the region led to structural adjustment programmes with reduced health funding and a stagnation and/or reduction in health outcomes.^3,4^ The HIV pandemic and persisting malaria burden among others led to a PHC approach that focused on HIV, TB and malaria under the Millennium Development Goals.^5^ While there was progress in this approach, overall health improvements were not commensurate with investments and led to an increasing focus on the need for a systems approach that eventually led to the focus on universal health coverage (UHC) from 2015 as part of the overall Sustainable Development agenda.^6^ In the WHO African Region, a common Regional Framework for Actions for health systems development to attain UHC and other health outcomes was agreed in 2017.^7^

The need to rethink the PHC approach based on the UHC focus, together with persisting outbreaks such as the West African Ebola Virus Disease outbreak and the need to respond to health determinants, gathered momentum since 2015. These efforts culminated in the 2018 Astana Declaration, which reaffirmed PHC as the cornerstone of health system development and proposed a revitalised implementation framework. This framework is operationalised through three categories of drivers. Representing the foundational principles of PHC, the first category includes PHC components: multisectoral action, community empowerment and integrated health services. The second category comprises strategic levers—political commitment and leadership, governance and policy frameworks, resource allocation and community engagement—which create an enabling environment for implementation. The third category comprises operational levers—including workforce, infrastructure, medicines and health products, models of care, quality improvement systems, digital health technologies, research, monitoring and evaluation, purchasing and payment systems and private sector engagement—which translate policy intentions into service delivery practice and measurable outcomes.^8^

At present, countries in the African region are prioritising both what to invest guided by the framework and how to invest guided by the revitalised PHC approach.^9–11^ Decision-makers are not sure how to link these two important lenses guiding health sectors. This is, in part, driven by a lack of clarity on how they should integrate the drivers of PHC within their health sectors and how to prioritise them. For this study, the revitalised PHC approach is conceptualised through three interrelated categories of drivers. First, the PHC components—multisectoral action, community empowerment and integrated health services—represent the foundational principles of PHC. Second, the strategic levers—political commitment and leadership, governance and policy frameworks, resource allocation and community engagement—create the enabling environment for PHC implementation. Third, the operational levers—including models of care, workforce, infrastructure, medicines and health products, quality improvement systems, digital health technologies, research, monitoring and evaluation, purchasing and payment systems and private sector engagement—translate policy intent into service delivery practice (table 1). Together, these drivers influence how health system investments are organised and used to improve functionality and ultimately achieve desired health outcomes.

**Table 1:** Definitions

Term	Definition
Revitalised PHC	The renewed PHC approach articulated in the Astana Declaration aimed at accelerating UHC, health security and health outcomes
PHC components	Multisectoral action, community empowerment and integrated health services
Strategic levers	Governance, leadership, policy frameworks, financing and community engagement mechanisms
Operational levers	Service delivery and system strengthening interventions that operationalise PHC
Health system investments	Financial, institutional, policy, technological and organisational inputs directed towards strengthening health systems
Health system functionality	The ability of health systems to provide accessible, quality, resilient and responsive services irrespective of the way it is organised
Health results	Improvements in UHC, health security, determinants of health and population health outcomes

Source: Authors’ own definitions of key terms used in this paper.

PHC, primary healthcare; UHC, universal health coverage.

While these different constructs of the revitalised PHC approach drivers have been well defined, there is limited evidence on their consolidated status, plus how they interact to influence health outcomes in country health systems. This therefore makes it difficult for health leaders to prioritise drivers and/or constructs as they apply the revitalised PHC approach in their health sectors. This paper explores this, presenting health leaders with a status and guidance on how to apply these different constructs based on their country situation.

## Methods

### Study design

This study employed a descriptive mixed-methods design to examine the relationship between the drivers of the revitalised PHC approach and the health systems and services required to achieve desired health outcomes across the 47 countries of the WHO African Region. A standardised questionnaire was developed and administered, capturing both policy intentions and implementation realities, enabling comparative analysis (see online supplemental appendix 1). The study was conducted across countries in the WHO African Region. Countries were requested for their consent to take part in the study, with email affirmations considered adequate.

### Conceptual framework

We examined the status and relationship between the drivers of the revitalised PHC approach across the health results chain, from implementation to impact. The results chain is defined in the regional framework of actions, detailing the relationship between investments, functionality and results (outcomes and impact) of health actions.^7^

For this study, health system investments refer to the resources, capacities and interventions directed towards strengthening the core domains of the health system. These include investments in the health workforce, health infrastructure, medicines and health products, health financing systems, governance arrangements, service delivery processes and health information systems. Such investments may take financial, institutional, policy, technological or organisational forms.

Health system functionality refers to the extent to which these investments collectively enable the health system to perform its intended functions effectively. Consistent with the WHO African Region Framework for Health Systems Development, functionality is assessed through four dimensions: access to services, quality of services, demand for services and system resilience. Functionality therefore reflects how well investments are translated into operational performance and service delivery outcomes.

**Figure 1:**
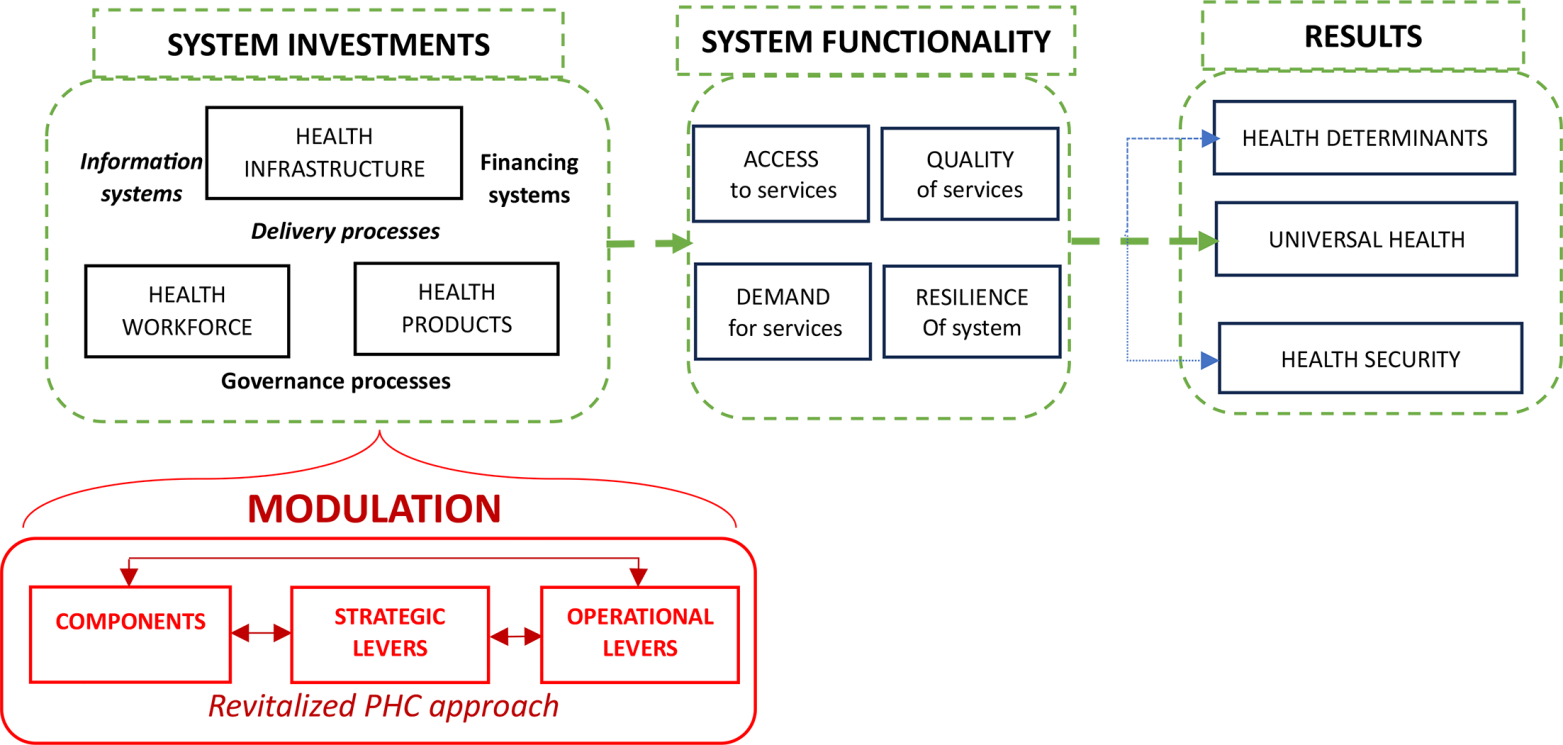
Applying the PHC approach to health systems and services outcomes. Source: Authors’ construction. PHC, primary healthcare.

Functionality is viewed as a measure of normative outputs all systems strive to attain, irrespective of the scale and interactions of investments they make across their building blocks. We presume that the revitalised PHC drivers (Strategic Enablers: Political Commitment, Governance and Policy, Funding and Community Engagement; and Operational Enablers: Models of Care, PHC Workforce, Physical Infrastructure, Medicines and Health Products, Private Sector Engagement, Purchasing and Payment Systems, Digital Health Technologies, Systems for Improving Quality of Care (QoC), PHC Oriented Research and Monitoring and Evaluation) have a modulation effect on the system investments made, facilitating the attainment of the desired level of functionality that gives the system the results it needs (see figure 1).

In this study, the term ‘drivers’ refers to the factors that influence how effectively PHC is implemented and translated into health system performance and health outcomes. The PHC components establish the core values and principles of implementation; strategic levers shape policy and governance environments; and operational levers determine how resources and investments are transformed into functional services. Together, these drivers influence the ability of health systems to achieve improved access, quality, resilience, health security and UHC.

The conceptual pathway examined in this study assumes that PHC components establish the foundational values and principles for health system development. These components shape strategic levers that influence policy direction, governance arrangements, resource allocation and stakeholder engagement. Strategic levers subsequently influence the nature and effectiveness of health system investments. Operational levers then determine how effectively these investments are translated into service delivery processes and system functionality. Improved functionality—characterised by enhanced access, quality, demand and resilience—is expected to contribute to improved health results, including UHC, health security and better determinants of health. The pathway therefore represents a sequence of reinforcing relationships linking PHC implementation to health outcomes.

The distinction between investments and functionality is important. Investments represent the inputs and resources that countries mobilise for health system strengthening, while functionality represents the performance generated from those investments. For example, investments in workforce training, infrastructure, medicines, financing mechanisms and information systems contribute to improved access, higher QoC, increased service utilisation and greater resilience to shocks. These dimensions of functionality subsequently influence broader health results, including UHC, health security and improved determinants of health.

The PHC drivers are not investments per se, but modulate and shape health investments to optimise their effect on health results.^11^ A strategic application of the revitalised PHC approach is therefore important as it enhances the use of existing health investments to create the needed health results in a country. We identify measures to quantify the description of the revitalised PHC approach drivers, plus the system and services to decipher this modulating relationship.

### Data collection

There is no routinely available data that covers this scope across all 47 countries of the WHO African region. To address this gap and ensure comparable data on PHC drivers and health systems across the region, we collected primary data from key stakeholders knowledgeable about the different drivers of the revitalised PHC approach in each country of the region.

WHO country office teams in each country purposively identified senior decision-makers from government, donors and partners involved in the revitalised PHC implementation process. These key informants (KIs) were required to have a national-level remit for PHC policy or implementation, hold their current role during the field period and be familiar with service delivery across regions, service areas, age groups and vulnerable populations. Individuals with strictly subnational or narrowly program-specific roles were excluded.

To minimise participant bias, each country aimed to include at least three KIs to balance perspectives of three constituencies: (1) government: a Ministry of Health official with national PHC/system responsibilities; (2) donor/implementing partners: a senior health advisor from a major in-country partner engaged in PHC and (3) a staff member supporting national PHC implementation or systems strengthening. 153 named key stakeholders—ranging from 1 to 9 per country—took part from 46 of the 47 countries (online supplemental appendix 2). To reduce potential respondent and institutional bias, participating countries were requested to identify stakeholders from different constituencies involved in PHC implementation, including government, development partners and implementation support organisations. This approach should capture diverse perspectives and reduce reliance on a single institutional viewpoint. Prior to data collection, all respondents received standardised guidance on the objectives of the assessment, definitions of key concepts and interpretation of the scoring framework to promote consistency in responses across countries.

### Instrument design

To ensure comparable data, we used a structured tool to collect KIs’ views based on attributes relating to the different drivers. Respondents scored these using a standard scale to separately capture both intention and practice, separating wishes from reality. The tool collected views on the 17 drivers of the PHC approach, together with information on the health results chain. For each driver, an attribute was defined that specified the expected status in the country. The KIs then scored their country based on their knowledge using a 6-point scale based on its (1) geographical distribution, (2) essential services provided across the care continuum, (3) age cohorts covered and (4) vulnerable populations reached (see online supplemental appendix 3). The KIs ensured that the country’s position was adequately represented. A 6-point scale was used to avoid clustering of responses in the middle. All areas were mandatory for each KI.

### Participation

Nominated KIs received an online orientation in October 2023 covering instrument purpose, definitions and anchors. They then completed the questionnaire via a secure electronic form during the field window (1 November 2023–29 February 2024). Where more than one stakeholder per category was nominated, all eligible responses were accepted; country-level indices were computed by aggregating ratings as described in the Analysis section. Quality checks for completeness and internal consistency were performed prior to analysis.

Health impact data in the results chain have publicly available comparable data for each country. As such, we used the WHO Global Health Observatory (WHO GHO) and included data on Health Security Index (HSI), UHC, Healthy Life Expectancy (HALE), State Party Annual Reporting (SPAR), health workforce densities, patient safety scores, QoC, user experience, political indices, poverty rates (inverse), childhood stunting rates (inverse) and broader social determinants of health.^12^ Other variables were the GGHE-D (Domestic General Government Health Expenditure) as % of GDP, GGHE-D as % of general government expenditure (GGE) and current health expenditure (CHE) collected to validate key health indices and assess government commitment to health financing.^12^

### Data preparation

Primary data responses were extracted from the online tool. The geometric mean of the multiple key stakeholders in each country was used to derive an average value for each attribute in each country. Likert scale responses were normalised to a scale between 0 and 1 for consistency. Secondary data from the WHO GHO were already processed and ready for analysis. Secondary data sources describing the WHO GHO indicators used for triangulation, their periodicity and the exact download date aligned to the survey field window.

### Analysis plan

The analysis focused on summarising data, validating findings and examining relationships within the PHC process.

### Data summarisation

Country-level geometric mean normalised scores for the PHC drivers, the health system investments and functionality variables were summarised as values between 0 and 1—with 0 being furthest from the desired attribute description and 1 closest to it. The geometric mean was preferred since the data were bound, and it is less sensitive to outliers than the arithmetic mean. The normalisation used the min-max normalisation approach (score–min)/(max–min) resulting in the bounded data (0–1). Associations between variables were computed using Spearman’s rank correlation to account for any non-normality and skewness in the realised scores with no further adjustments. Differences in performance scores were assessed using one-way analysis of variance (ANOVA) across four categories: country income level, population density, land type (Small Island States, Landlocked, Non-Landlocked) and land area size.

### Relationship analysis

The pathway analysis examined how different PHC drivers are associated with health system investments, functionality and results. In this study, a pathway refers to a statistically supported relationship between two or more components of the PHC implementation framework. The pathways were derived empirically using structural equation modelling (SEM), which enabled a simultaneous assessment of direct and indirect relationships among PHC drivers, health system functionality and health outcomes. The resulting pathways should therefore be interpreted as evidence-informed implementation relationships rather than deterministic or causal sequences.

To determine the pathways for linking the revitalised PHC drivers and the health system investments, we explored these relationships using regression models for each of the constituent drivers—PHC components, strategic levers, operational levers—to identify the PHC drivers that best explain the health systems investments using coefficients of determination. The PHC progress pathways were conceptualised using a SEM, enabling the simultaneous analysis of multiple independent, intermediate and dependent variables. Given that many concepts are unobservable, they were measured indirectly using the scores of their individual attributes. For example, the PHC components were assessed through multisectorality, empowerment and integration, based on coverage in geographical areas, services, age cohorts and vulnerable populations. This method provides a more precise measurement of key theoretical concepts.

The model framework involved latent and observed variables that were built in both measurement and structural models. The fitted measurement model was:


$$Y=\Lambda_{y}\psi +\varepsilon$$

where

Y=a vector of observed variables (eg, survey responses like UHC index, HSI and determinants of health for Results).

Λ=matrix of factor loadings, showing how much each latent variable contributes to the observed variables.

ψ=vector of latent variables (eg, Results);

ε=vector of measurement errors, accounting for the variance in observed variables not explained by the latent variables.

The structural model was:


$$\psi =B_{\psi }\eta +\xi$$

where

ψ=vector of endogenous latent variables (ie, dependent variables);

η=vector of exogenous latent variables (ie, independent variables);

Β=matrix of path coefficients that shows the relationships between the latent variables;

ξ=vector of structural errors, accounting for the variance in the endogenous variables not explained by the exogenous variables.

The modelling approach was stepwise, designed to isolate the effects of different components and address any confounding using SEM. Model 1 is the baseline pathways model with functionality as the mediator between investments and results. It tests the impact of functionality as a mediator, while also testing the direct investment to results path to assess whether any association remains after accounting for functionality. Model 2 extends the relationship above by independently exploring the impact of the PHC components, strategic levers and operational levers individually on the results chain. Finally, model 3 brings together all the relationships defined in the two models based on the areas where the relationships were strongest to generate a unified causal chain. This creates a comprehensive partial least squares SEM (PLS-SEM) framework, focusing on the strength of relationships across all the variables. The paths with the strongest relationships represent the best value, while relatively weaker or negative relationships are not recommended.

### Internal consistency and model validation

Validity checks were conducted to identify inconsistencies within the data. Spearman’s correlation applied to test expected positive and significant associations based on our hypothesis, including relationships between PHC components and health system investments, health system investments and functionality and functionality and health results, along with other hypothesised health outcome associations (online supplemental appendix 4).

To ensure internal consistency and model validation, goodness-of-fit was assessed using Cronbach’s alpha, rhoC and rhoA, each evaluated against a reliability threshold of 0.7 to confirm construct reliability.^13^ Average variance extracted (AVE) was examined against a threshold of 0.5 to validate latent constructs. These metrics were used to check the model’s robustness to ensure its ability to capture meaningful relationships within the PHC framework.

Although the study was not specifically designed to assess inter-rater reliability, several approaches were employed to strengthen confidence in the stakeholder assessments. These included the use of multiple respondents per country, representation from different stakeholder constituencies, aggregation of responses using geometric means and triangulation against independent external indicators. Construct reliability and validity were further assessed using Cronbach’s alpha, rhoA, rhoC and AVE, all of which met accepted thresholds for reliability and validity.

### External validation of indices

The primary indices of the revitalised PHC approach drivers and the health results chain are from the primary data. To assess their credibility, we examined whether they showed expected monotonic associations with independent country datasets that represent related constructs (convergent/criterion-related validity). Thus, the indices were correlated with public indicators from the WHO GHO: HSI, UHC, HALE, SPAR, health-workforce densities, patient safety/quality, user experience, political indices, poverty, stunting and other social determinants, and with health-financing variables (GGHE-D as % GDP and % GGE, CHE). Spearman’s rank correlations were computed between the indices and the external indicators because the primary measures originate from ordinal Likert items and the aggregated country scores are bounded and potentially non-normal. Some of the hypothesised pairings were level of PHC implementation and health financing.

The PHC implementation indices developed in this study are intended as relative measures of the extent to which countries have implemented the drivers of the revitalised PHC approach. The analysis assumes that the conceptual definitions and measurement constructs were interpreted consistently across countries, following standardised orientation and guidance provided to respondents. While efforts were made to maximise comparability through the use of common instruments, structured scoring criteria and external validation against independent datasets, implementation of PHC remains inherently context-specific. The resulting scores should be interpreted as indicative measures of relative implementation status rather than absolute measures of health system performance or effectiveness. Differences in governance arrangements, health system organisation, resource availability and sociopolitical contexts may influence both implementation and stakeholder perceptions.

### Sensitivity analysis

Sensitivity analysis was conducted using SEM to systematically evaluate the contribution of each component to the overall pathway model. Individual components, such as strategic and operational levers and system investments, were assessed separately within distinct models based on model 1, allowing for an independent evaluation of their impact using coefficients of variation.

Variables were then introduced sequentially, each building block of the independent variable at a time while checking for the additional variation explained, gradually expanding from simpler models to the full framework, refining the assessment of their interconnections. The full model included all components but also contained trivial paths, helping identify the most meaningful relationships driving health system functionality and outcomes.

### Bootstrapping in SEM analysis

To assess the stability and reliability of the parameter estimates in the SEM, bootstrapping procedures were employed, as they provide robust inference without relying on parametric distributions. It was done to generate multiple sub-samples from the original dataset to estimate the precision of model parameters, including path coefficients, SEs and t-statistics. For efficiency of estimation, we generated 1000 bootstrap samples to evaluate the significance of the structural paths. The analysis was conducted using the SEMinR package (V.2.3.0) in R, which supports bootstrapping for PLS-SEM and provides detailed diagnostics for model robustness.^14^

## Results

Significant correlations were observed between the primary data, in related data domains and the external data sets. For instance, all the economic indicators had a significant positive correlation with the operational levers. This was also the case with Human Resources for Health (HRH) index and the nurses and midwives density per 10 000 population. The health index values also had strong correlations with the positivity of the survey questions. These different external datasets validated the informed opinion to a great extent, hence increasing the confidence with which the data were used to conduct the study (online supplemental appendix 4).

When we explored the reliability of the model parameters, all were adequate and reliable in model 3, with alpha, rhoC and rhoA values for all the primary data exceeding 0.7 (online supplemental appendix 5).

Scores are presented for practice and not for intention. The index for intention was higher by 26% than practice for the operational levers and 23% for the strategic levers, though this varied by variable and country (online supplemental appendices 6 and 7). Only four countries—Algeria, Niger, Ethiopia and Burkina Faso—had non-significant variations between strategic levers intention and practice, while the widest gaps were with the Democratic Republic of Congo, Guinea Bissau, Ivory Coast, Mauritania, Chad and Madagascar (online supplemental appendix 8).

**Figure 2:**
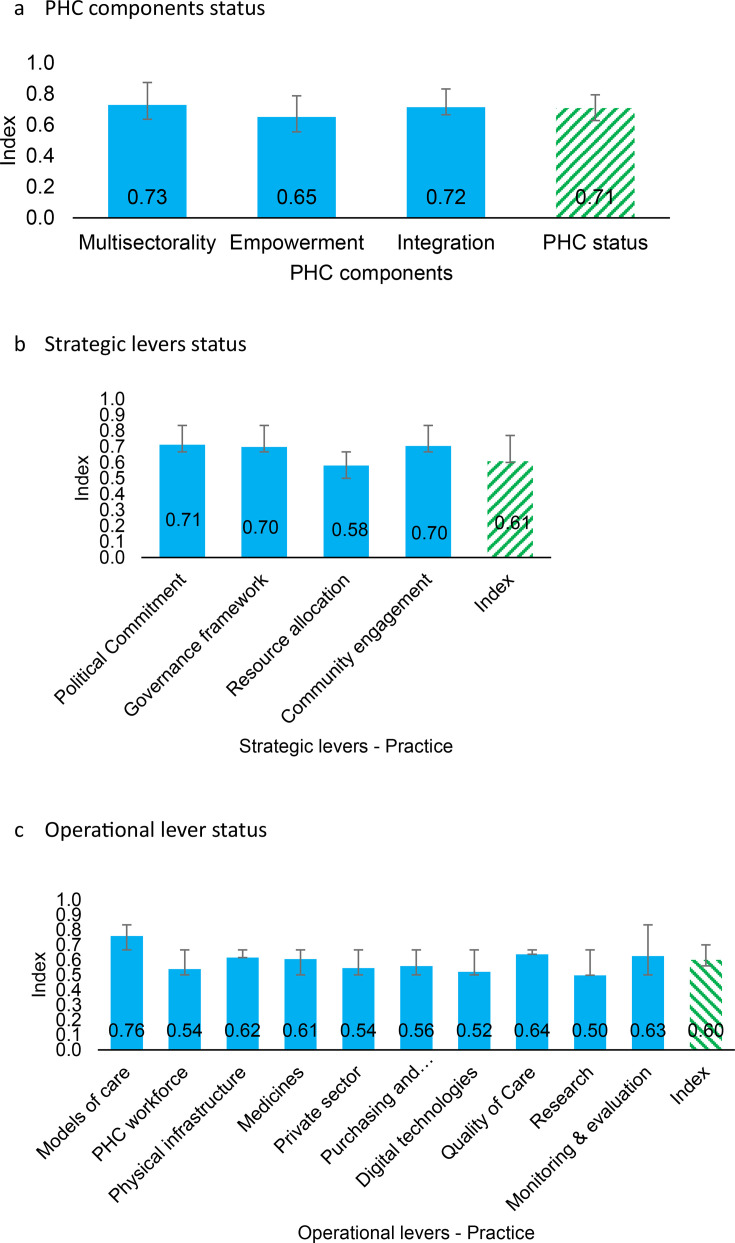
The state of the PHC approach in the WHO African Region for a) PHC component, b) strategic lever and c) operational lever

The overall geometric mean for practice for revitalised PHC approach is shown in figure 2. The mean PHC index score for the region is 0.66, with the score for PHC components highest at 0.71, followed by strategic levers at 0.67, while operational levers score 0.60. The values for each are driven by significant differences in the values for their constituent variables. For instance, the PHC components score has the multisectorality variable highest at 0.73 (Q1: 0.64, Q3: 0.88 across the countries), followed by the integration variable at 0.72 (Q1: 0.67, Q3: 0.83) while empowerment averaged 0.65 (Q1: 0.56, Q3: 0.79) (online supplemental appendices 8 and 9).

The results by country are shown in table 2. The revitalised PHC approach score was highest in South Africa, scoring 0.90 and lowest for Sao Tome and Principe at 0.37. 26 countries score above the regional mean, with 20 countries below it.

**Table 2:** The status of PHC implementation and the levers across the WHO African Region

Position	Country	PHC index (mean score)	PHC components scores	Strategic levers scores	Operational levers scores
1	South Africa	0.90	1.00	0.89	0.81
2	Benin	0.89	1.00	0.94	0.74
3	United Republic of Tanzania	0.88	0.99	0.87	0.77
4	Rwanda	0.83	0.89	0.85	0.75
5	Uganda	0.82	0.80	0.87	0.80
6	Zimbabwe	0.81	0.88	0.83	0.71
7	Ghana	0.78	0.74	0.87	0.74
8	Zambia	0.77	0.85	0.74	0.72
9	Kenya	0.76	0.67	0.84	0.78
10	Mauritius	0.76	0.86	0.73	0.69
11	Ethiopia	0.76	0.75	0.77	0.75
12	Senegal	0.75	0.78	0.88	0.60
13	Togo	0.75	0.89	0.77	0.59
14	Eswatini	0.75	0.83	0.71	0.71
15	Sierra Leone	0.75	0.94	0.71	0.59
16	Burkina Faso	0.75	0.72	0.83	0.69
17	Malawi	0.73	0.77	0.78	0.63
18	Namibia	0.71	0.74	0.76	0.63
19	Niger	0.71	0.74	0.67	0.71
20	Cabo Verde	0.70	0.81	0.67	0.63
21	Eritrea	0.70	0.70	0.73	0.67
22	Ivory Coast	0.69	0.78	0.62	0.66
23	Seychelles	0.69	0.70	0.73	0.63
24	Angola	0.68	0.71	0.68	0.65
25	Comoros	0.68	0.71	0.71	0.61
26	Burundi	0.67	0.72	0.67	0.62
27	Mauritania	0.65	0.75	0.57	0.64
28	Botswana	0.64	0.70	0.62	0.60
29	Algeria	0.64	0.67	0.61	0.64
30	Liberia	0.64	0.61	0.69	0.61
31	Gambia	0.63	0.71	0.68	0.51
32	Nigeria	0.63	0.59	0.69	0.60
33	Equatorial Guinea	0.62	0.73	0.63	0.51
34	Gabon	0.60	0.61	0.62	0.57
35	Chad	0.59	0.79	0.60	0.38
36	Lesotho	0.59	0.61	0.54	0.61
37	Mozambique	0.58	0.55	0.65	0.55
38	Guinea	0.54	0.56	0.47	0.60
39	Central African Republic	0.54	0.65	0.49	0.47
40	Mali	0.53	0.63	0.41	0.56
41	Madagascar	0.52	0.53	0.50	0.53
42	Democratic Republic of Congo	0.52	0.53	0.54	0.49
43	South Sudan	0.51	0.64	0.50	0.39
44	Cameroon	0.50	0.56	0.51	0.44
45	Guinea Bissau	0.42	0.43	0.46	0.36
46	Sao Tome and Principe	0.37	0.34	0.51	0.27

PHC, primary healthcare.

PHC components index ranges from 0.34 (Sao Tome and Principe) to 1.00 (Benin and South Africa), with 21 countries scoring below the regional mean and 26 above average. The strategic levers score varies from 0.41 (Mali) to 0.94 (Benin), with South Africa (0.89), Senegal (0.88) and Ghana (0.87) demonstrating strong scores. The operational levers score ranges from 0.27 (Sao Tome and Principe) to 0.81 (South Africa), with Uganda (0.80), Kenya (0.78) and Tanzania (0.77) showing robust operational lever scores. The scores reflect the relative implementation of PHC drivers rather than absolute health system performance.

One-way ANOVA revealed no statistically significant differences in PHC Index based on country size (F=0.216, p=0.807; large, medium, small), population density (F=1.332, p=0.275; low, medium, high tertiles), income category (F=0.873, p=0.462; high, upper-middle, lower-middle, low) or land type (F=0.705, p=0.500; Small Island States, landlocked, non-landlocked states). This non-significance was not only for the overall PHC index but also for its constituents: PHC components, strategic levers and operational levers (see online supplemental appendix 10 for category definitions). However, a consistent descriptive trend was noted: small Island States (mean PHC index=0.620) had a lower index than landlocked (mean PHC index=0.688) and coastal nations (mean PHC index=0.675).

**Figure 3:**
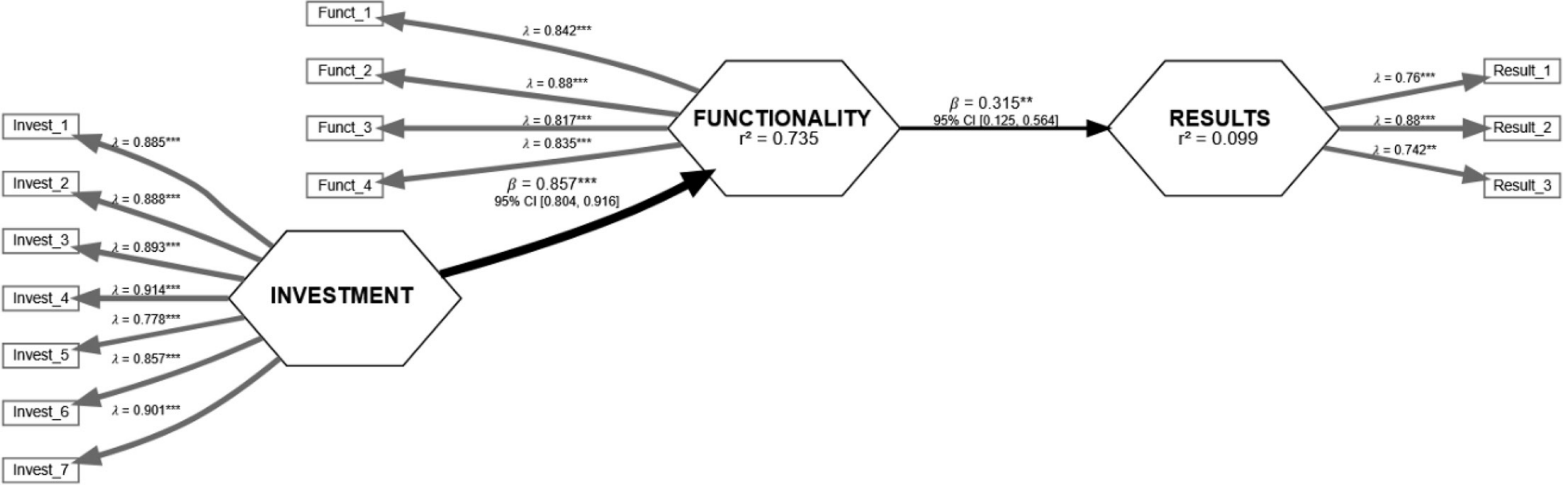
Structural model 1 illustrating the pathway relationships among investment, functionality and health outcomes. Key:p < 0.01 = “**”, p < 0.001 = “***”

Model 1 results are shown in figure 3. Bootstrapping stability confirmed the reliability of the estimates, with high T-statistics (28.516 and 2.549) and small SEs supporting model robustness. The path analysis revealed a strong relationship between investment and functionality (β=0.857 (95% CI 0.804 to 0.916), p<0.001), explaining 74% of the variance in system functionality (R²=0.735). The pathway from Functionality to Results exhibited a moderate but significant effect (β=0.315 (95% CI 0.125 to 0.564), p=0.011), with a low explanatory power (R²=0.099), suggesting that additional factors beyond functionality are needed to achieve optimal health results. The small SEs support the model robustness.

Reliability assessments showed excellent internal consistency for Investment (α=0.949), strong reliability for Functionality (α=0.865) and acceptable reliability for Results (α=0.718). All constructs surpassed the 0.7 threshold, validating their reliability. Convergent validity was confirmed, as AVE exceeded 0.5 across constructs.

Model 2 results show the impact of introducing the different PHC drivers (online supplemental appendix 11); the PHC components (2a), strategic levels (2b) and operational levers (2c).

Model 2a (online supplemental appendix 11)—inclusion of the PHC components to the investments-functionality-results pathway shows a sequential pathway wherein the PHC components significantly drive investment (β=0.664 (95% CI 0.448 to 0.817), p<0.001, R²=0.441), which fosters system functionality (β=0.857 (95% CI 0.797 to 0.916), p<0.001, R²=0.734). However, the link between functionality and health outcomes is comparatively modest (β=0.315 (95% CI 0.125 to 0.564), p=0.011, R²=0.099), showing that while investments yield highly functional systems, additional factors may be essential to fully realise improved health outcomes. Reliability and validity assessments across all constructs are strong.

Model 2b (online supplemental appendix 11)—inclusion of strategic levers to the investments-functionality—results pathway shows a stronger influence on investment than model 2a (β=0.728 (95% CI 0.595 to 0.84), p<0.001, R²=0.529), demonstrating strategic levers appear more critical for impacting on investments. The impact of investments on functionality remains similarly strong as with model 2a (β=0.856 (95% CI 0.798 to 0.915), p<0.001, R²=0.732), and that of functionality to results also remains similarly positive but modest (β=0.315 (95% CI 0.125 to 0.564), p=0.011, R²=0.099).

Model 2c (online supplemental appendix 11)—inclusion of operational levers to the investments-functionality—results pathway shows the strongest influence on investments (β=0.806 (95% CI 0.681 to 904), p<0.001, R²=0.650), with the investment-functionality pathway remaining equally strong (β=0.857 (95% CI 0.797 to 0.915), p<0.001, R²=0.734) and the functionality-results pathway remaining positive but modest (β=0.315 (95% CI 0.125 to 0.564), p=0.011, R²=0.099).

**Figure 4:**
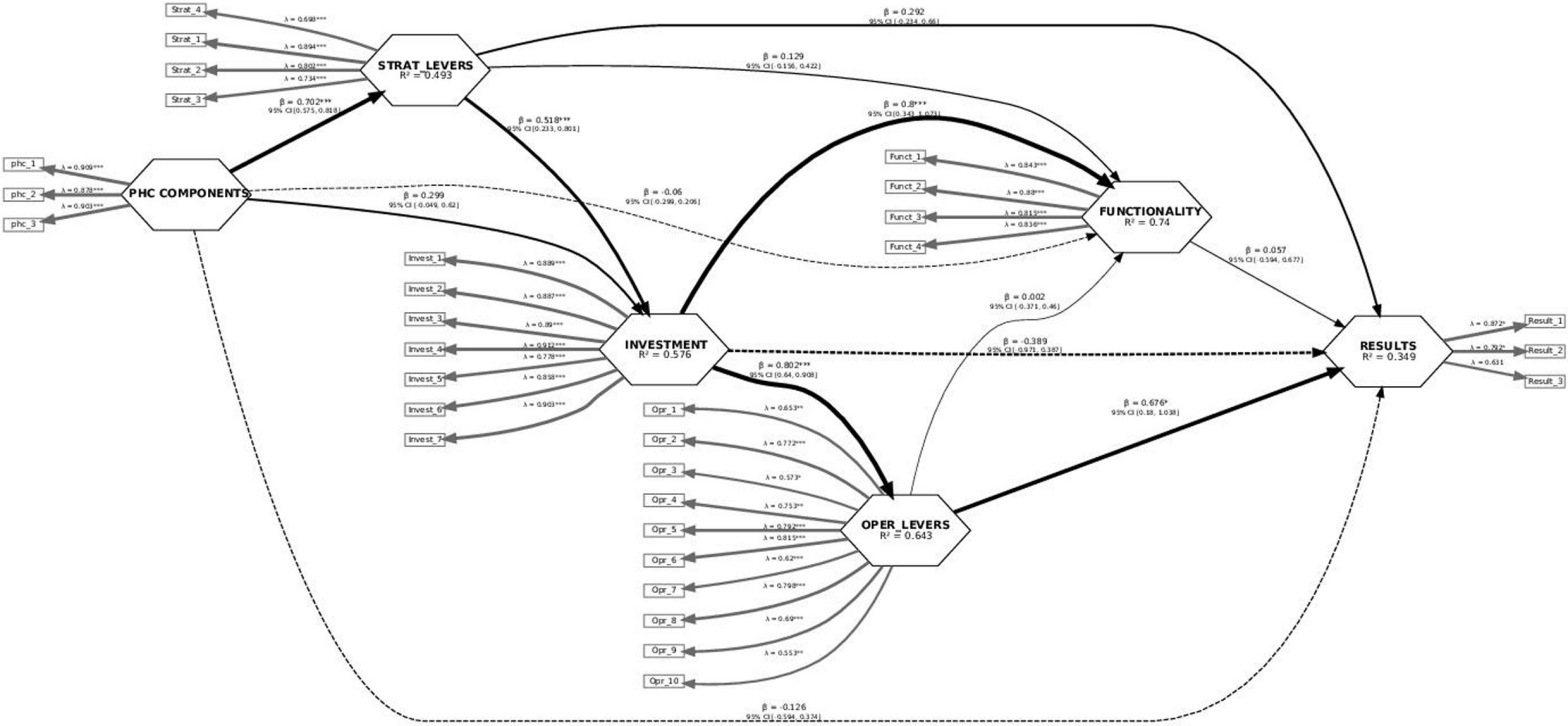
Impact of all revitalised PHC approach drivers on the investment-functionality-results pathway. Key: < 0.05 = “*”, p < 0.01 = “**”, p < 0.001 = “***”

Model 3 now integrates all the three revitalised PHC drivers on the investment-functionality-results pathway, guided by model 2 results (see key for models 1–3 in online supplemental appendix 12). The resulting structural model is shown in figure 4. The strongest influences are from PHC components to strategic levers (β=0.702 (95% CI 0.562 to 0.817), p<0.001) and from strategic levers to investments (β=0.518 (95% CI 247 to 0.774), p<0.001). However, from investments, influences are equally strong to operational levers (β=0.802 (95% CI 0.666 to 0.905), p<0.001) and to functionality (β=0.800 (95% CI 0.666 to 1.091), p<0.001). However, operational levers show a much stronger influence on results (β=0.676 (95% CI 0.12 to 1.031), p<0.01) than functionality (β=0.057 (95% CI −0.569 to 0.679), p=0.86).

Additional, but less significant pathways are also seen. For instance, negative predictions are seen between PHC components and functionality (β=−0.060 (95% CI −0.298 to 0.198), p>0.05), PHC components and results (β=−0.126 (95% CI −0.619 to 0.417), p=0.62) and investments and results (β=−0.389 (95% CI −1.005 to 0.427), p=0.27). Other predictions are weaker than those described in the optimal pathway.

## Discussion

This paper developed and applied a multicountry approach to assess countries’ progress in implementing the revitalised PHC approach and identify optimal pathway models for its implementation. This study examined both the status of implementation of the revitalised PHC approach and the pathways through which its drivers contribute to health system performance and health outcomes. Conceptually, the findings suggest that PHC components provide the foundational principles for implementation, strategic levers create the enabling environment for decision-making and investment and operational levers translate these investments into service delivery improvements. Understanding these interactions is critical for countries seeking to strengthen PHC as a pathway to UHC, health security and improved population health outcomes.

A central contribution of this study is the demonstration that PHC implementation is not driven by individual interventions in isolation but by interactions among multiple PHC drivers operating across the health system. The findings suggest that PHC components provide the foundational principles for implementation, strategic levers create the enabling environment for investment and governance, and operational levers translate these investments into improvements in service delivery and health system performance. Understanding these interactions is critical for countries seeking to strengthen PHC as a pathway towards UHC and health security.

An important distinction emerging from this study is that investments alone do not guarantee improved health outcomes. Rather, the effectiveness with which investments are translated into functionality—through improved access, quality, demand and resilience—determines their ultimate contribution to health results. This finding reinforces the importance of focusing not only on the volume of investments but also on how they are organised, governed and implemented within health systems.

The overall 26% gap between the intended goals of PHC policies and their actual implementation quantifies misunderstanding between what people intend to do and what is happening. All countries where the gaps were most significant are low-income countries, plus countries with some levels of past or current conflict. This highlights the importance of not only looking at what is stated but exploring what is being implemented in a country with complex needs.

Interestingly, the revitalised PHC approach score was not influenced by income or country size, challenging the assumption that higher spending guarantees better performance. Coastal and higher-density regions outperformed Small Island States and landlocked areas, likely reflecting a set of structural and contextual factors. For the Small Island States, this could reflect their greater exposure to unpredictable climatic events, the reliance of other countries for and development of critical system inputs such as their HRH and digital ecosystem, all of which affect multiple health system pillars simultaneously.^15^ Looking at PHC components, implementation was strongest for multisectorality, followed by integration and weakest for empowerment. This pattern underscores that the health sector alone cannot achieve optimal results; progress depends on cross-sector coordination, effective service integration and genuine community involvement. The relatively low score for community engagement is striking, given the focus this area has had since the beginning of the PHC approach. This may stem from the conflation of community engagement or participation with genuine empowerment during implementation. In practice, many initiatives reported as community empowerment still centre on service delivery or peer education, with few creating space for communities to influence decisions or policy.^15,16^ Building the capacity of individuals and families to act in the interests of their own well-being would shift the region from viewing communities as passive recipients of care to recognising them as active agents with self-defined needs.

Among strategic levers, resource allocation shows the least progress. Evidence from regional analyses shows that financing remains concentrated on commodities with limited investment in human resources, governance and infrastructure.^17^ These imbalances suggest resources are not being distributed across the full range of system needs. These domestic imbalances are exacerbated by external misalignments. Studies have shown that international aid often diverges from disease burden and national income, with some countries receiving substantially more or less aid than expected.^18^ Global health initiatives have sometimes skewed national priorities by emphasising specific diseases and creating parallel systems.^19^ These patterns underscore that increased funding alone is insufficient; progress depends on deliberate, evidence-informed allocation aligned with national priorities.

Another important finding is the critical role that operational levers play in the PHC implementation. They ensure investments are effectively managed and translated into functional and high-quality services, and act as a bridge between investment and impact. The modelling results show operational levers influence both system functionality and health results. This suggests that addressing operational levers provides a more direct pathway to tangible outcomes than originally hypothesised in the investment-functionality-results chain. This pattern is illustrated with Benin, which ranks high in our results and represents an interesting finding. Evidence from the health system functionality analysis shows that Benin performs well in the quality and demand dimensions of system functionality, despite weaker performance in resilience.^11^ This suggests that prioritising the PHC approach yields early gains in service quality and utilisation, even where broader system resilience remains constrained.

In our findings, we notice that progress remains modest in research, digital technology and in both the PHC workforce and private-sector engagement. Although some countries like Nigeria, Kenya and South Africa have advanced national research systems, they still face limited coordination and heavy dependence on external funding.^20,21^ This gap significantly affects the translation of evidence into health policy among the countries and limits evidence-informed decision-making. As a result, policy responses often become unpredictable and potentially misaligned with actual needs.

Digital transformation, while widely endorsed, continues to be constrained by weak infrastructure and limited interoperability.^22,23^ Health workforce shortages persist across all levels of care, and private-sector engagement, though expanding, remains fragmented and weakly regulated.^24,25^ Strengthening these areas will be essential for translating investment into results and advancing the revitalised PHC agenda in the region.

Ultimately, the optimal pathway derived from this analysis reinforces that success lies not only in adopting the revitalised PHC approach but in structuring and sequencing its drivers and constructs to maximise impact on health results. It is common for sectors to focus on specific systems and services, without appreciating the preceding and/or proceeding components that need to be focused on too, to maximise health results.

### Limitations

This study’s strengths include its region-wide scope, structured key-informant data from the stakeholders, and triangulation with independent indicators. In terms of limitations, this study relies on comparable multicountry key-informant perceptions, which, although structured and anchored, remain subject to potential selection and positional bias. Three senior stakeholders were sampled per country and standardised prompts were used to limit these biases. Primary indices were triangulated against independent indicators (UHC Service Coverage Index, HALE, SPAR, workforce density, quality and safety metrics and sociopolitical indices) to reduce common-method concerns. One country did not take part; no item-level missingness occurred in the returned questionnaires. Because indices are bounded and partly ordinal, min–max normalisation and geometric means were applied for country aggregation. For latent constructs, internal consistency and convergent-validity metrics were reported, and non-parametric bootstrap inference in PLS-SEM was used to enhance robustness. External indicators were aligned with the survey window; where data lagged, the nearest year was used and weak or non-significant associations were interpreted cautiously. The time lag for the external data to the survey was tolerable since they were at outcome and impact levels that are expected to change less frequently within a brief time span. Cross-cultural interpretation of items may vary despite explicit anchors. Future research should focus on tool calibration through country case-studies, integration of facility-level micro-data (quality, workforce, expenditure) and periodic re-fielding to assess responsiveness to reforms and track PHC progress.

## Conclusions

Taken together, these findings suggest that implementing the revitalised PHC approach does not follow a single linear pathway but depends on multiple, context-dependent routes shaped by how PHC components and drivers are structured and sequenced within each country. It is important for health systems and services planning and monitoring to focus not only on the investment-functionality-results chain but also on the revitalised PHC drivers for a more robust sector oversight. Exploration of the relative weights different drivers contribute would further strengthen the targeting of the PHC approach in countries. As such, the systematic and integrated application of the revitalised PHC drivers should contribute to more sustainable health outcomes across the African region.

## Supplementary material

10.1136/bmjph-2026-005396 online supplemental file 1

## Data Availability

Data are available in a public, open access repository. Data are available on reasonable request.
