# Supplementary material for: Status, and pathways to implementing the revitalised PHC approach in the WHO African Region: a mixed-methods study
Source: BMJ Public Health. 2026 Aug 7;4(3):e005396. doi: 10.1136/bmjph-2026-005396 (PMC13475399; doi:10.1136/bmjph-2026-005396)
Supplement: Supplementary file 1 [file bmjph-4-3-s001.pdf]

Appendices

Appendix 1: The study questionnaire 1
Appendix 2: List of participants by country and affiliation 20
Appendix 3: The constructs and their variables 22
Appendix 4: Spearman’s rank correlation between the primary data and the related secondary data 26
Appendix 5 : Reliability of the model parameter 30
Appendix 6: The percentage gap between intention and practice of the strategic levers 30
Appendix 7: The percentage gap between intention and practice of the operational levers 30
Appendix 8: The intention and practice of strategic levers to PHC 31
Appendix 9: The intention and practice of operational levers to the PHC approach 31
Appendix 10. Classification Criteria for Country Attributes 32
Appendix 11: Impact of individual revitalized PHC approach drivers on investment-functionality-results pathway 34
Appendix 12: The key of models 1, 2 and 3 35

Appendix 1: The study questionnaire

COMPONENTS						
Dimension	Description	**Country situation**<br>Defined by 4 parameters: (1) How widespread they are, (2) which services are involved, (3) which population groups are involved, and (4) how well they are reaching vulnerable populations. For each, choose a number that best relates to the country's situation for the specific components. The key to determining the country score is provided below:				Notes
		Geographical area covered: The description applies to every sub-national unit in the country. Sub-national units are the devolved units responsible for health management and could be a district/sub-district, health district, county, province, state or other description used in the country.<br><br>The key for determining country score:<br>(1) Very strongly disagree (no doubts at all)<br>(2) Strongly disagree (minor doubts)<br>(3) Disagree (some doubts, but still largely inaccurate)<br>(4) Agree (some doubts, but still largely accurate)<br>(5) Strongly agree (minor doubts)<br>(6) Very strongly agree (no doubts at all)	*Services covered*: The description applies to all the essential service areas in the country. Essential service areas are (1) promotive, (2) preventive, (3) diagnostic, (4) curative, (5) rehabilitative, and (6) palliative.<br><br>The key for determining country score:<br>(1) Very strongly disagree (no doubts at all)<br>(2) Strongly disagree (minor doubts)<br>(3) Disagree (some doubts, but still largely inaccurate)<br>(4) Agree (some doubts, but still largely accurate)<br>(5) Strongly agree (minor doubts)<br>(6) Very strongly agree (no doubts at all)	*Age cohorts covered*: The description applies to all the age cohorts in the country. Age cohorts include (1) maternal/newborn, (2) under 5's, (3) 6-9 year olds, (4) adolescents 10-19 years, (5) adults, and (6) the elderly.<br><br>The key for determining country score:<br>(1) Very strongly disagree (no doubts at all)<br>(2) Strongly disagree (minor doubts)<br>(3) Disagree (some doubts, but still largely inaccurate)<br>(4) Agree (some doubts, but still largely accurate)<br>(5) Strongly agree (minor doubts)<br>(6) Very strongly agree (no doubts at all)	*Vulnerable populations covered:* The description applies to all the vulnerable groups in the country. Vulnerabilities are due to (1) place of residence, (2) race/culture, (3) occupation, (4) gender/sex, (5) religion, (6) education, (7) socioeconomic status, and (8) social capital<br><br>The key for determining country score:<br>(1) Very strongly disagree (no doubts at all)<br>(2) Strongly disagree (minor doubts)<br>(3) Disagree (some doubts, but still largely inaccurate)<br>(4) Agree (some doubts, but still largely accurate)<br>(5) Strongly agree (minor doubts)<br>(6) Very strongly agree (no doubts at all)	

Multisectorality	There are health sector actions to ensure individuals and families are receiving services from other health-related institutions & sectors that impact their health and well-being. Other sectors include education, water, roads, etc					

Empowerment	There are health sector actions to ensure individuals and families can make decisions about the services they need for their health and well-being, and can also enforce these decisions					
Integration	There are health sector actions to combine different essential services in a way that holistically responds to the needs of individuals and families, not only as standalone programs.					

Dimension	Description of the attributes	Country situation (based on actions carried out in the past 2 years)<br>Defined by 2 parameters:<br>(A) What is the intent<br>(B) What is actually in place		Notes
		(A) The description correctly describes what is intended in the country (intention)<br>**1. Very strongly disagree (no doubts at all)**<br>**2. Strongly disagree (minor doubts)**<br>**3. Disagree (some doubts, but still largely inaccurate)**<br>**4. Agree (some doubts, but still largely accurate)**<br>**5. Strongly agree (minor doubts)**<br>**6. Very strongly agree (no doubts at all)**	***(B) The description correctly describes the situation in practice (actuality)***<br>**1. Very strongly disagree (no doubts at all)**<br>**2. Strongly disagree (minor doubts)**<br>**3. Disagree (some doubts, but still largely inaccurate)**<br>**4. Agree (some doubts, but still largely accurate)**<br>**5. Strongly agree (minor doubts)**<br>**6. Very strongly agree (no doubts at all)**	
**STRATEGIC LEVERS**				
Political commitment & leadership	The Government has explicitly indicated its willingness to the revitalized PHC approach as the means to attain Universal Health Coverage, Health Security and Health outcomes across the determinants			
Governance & policy frameworks	The country has updated its policy, strategic and legal frameworks in line with the revitalized PHC approach			

Dimension	Description of the attributes	Country situation (based on actions carried out in the past 2 years)<br>Defined by 2 parameters:<br>(A) What is the intent<br>(B) What is actually in place		Notes
		(A) The description correctly describes what is intended in the country (intention)<br>**1. Very strongly disagree (no doubts at all)**<br>**2. Strongly disagree (minor doubts)**<br>**3. Disagree (some doubts, but still largely inaccurate)**<br>**4. Agree (some doubts, but still largely accurate)**<br>**5. Strongly agree (minor doubts)**<br>**6. Very strongly agree (no doubts at all)**	***(B) The description correctly describes the situation in practice (actuality)***<br>**1. Very strongly disagree (no doubts at all)**<br>**2. Strongly disagree (minor doubts)**<br>**3. Disagree (some doubts, but still largely inaccurate)**<br>**4. Agree (some doubts, but still largely accurate)**<br>**5. Strongly agree (minor doubts)**<br>**6. Very strongly agree (no doubts at all)**	
Funding and allocation of resources	The country has updated its initiatives to increase resources, improve their management and introduce targeted purchasing of essential services to facilitate implementation of its revitalized PHC approach			

Dimension	Description of the attributes	Country situation (based on actions carried out in the past 2 years)<br>Defined by 2 parameters:<br>(A) What is the intent<br>(B) What is actually in place		Notes
		(A) The description correctly describes what is intended in the country (intention)<br>**1. Very strongly disagree (no doubts at all)**<br>**2. Strongly disagree (minor doubts)**<br>**3. Disagree (some doubts, but still largely inaccurate)**<br>**4. Agree (some doubts, but still largely accurate)**<br>**5. Strongly agree (minor doubts)**<br>**6. Very strongly agree (no doubts at all)**	***(B) The description correctly describes the situation in practice (actuality)***<br>**1. Very strongly disagree (no doubts at all)**<br>**2. Strongly disagree (minor doubts)**<br>**3. Disagree (some doubts, but still largely inaccurate)**<br>**4. Agree (some doubts, but still largely accurate)**<br>**5. Strongly agree (minor doubts)**<br>**6. Very strongly agree (no doubts at all)**	
Engagement of communities & other stakeholders	The country has clear processes to engage communities, partners and other stakeholders for the revitalized PHC approach			
**OPERATIONAL LEVERS**				

<table>
<tr><th rowspan="2">Dimension</th><th rowspan="2">Description of the attributes</th><th colspan="2">Country situation (based on actions carried out in the past 2 years)
Defined by 2 parameters:
(A) What is the intent
(B) What is actually in place</th><th>Notes</th></tr>
<tr><th>(A) The description correctly describes what is intended in the country (intention)
1. Very strongly disagree (no doubts at all)
2. Strongly disagree (minor doubts)
3. Disagree (some doubts, but still largely inaccurate)
4. Agree (some doubts, but still largely accurate)
5. Strongly agree (minor doubts)
6. Very strongly agree (no doubts at all)</th><th>(B) The description correctly describes the situation in practice (actuality)
1. Very strongly disagree (no doubts at all)
2. Strongly disagree (minor doubts)
3. Disagree (some doubts, but still largely inaccurate)
4. Agree (some doubts, but still largely accurate)
5. Strongly agree (minor doubts)
6. Very strongly agree (no doubts at all)</th><th></th></tr>
<tr><td>Models of care</td><td>The country is planning, and using different models to provide the full range of essential services. These are:
Primary care level: A core primary care facility, community units, mobile services, community clinics, community units, medical/surgical camps, community-owned resource persons
Hospital level: Primary, secondary, and tertiary hospitals</td><td></td><td></td><td></td></tr>
</table>

Dimension	Description of the attributes	Country situation (based on actions carried out in the past 2 years)<br>Defined by 2 parameters:<br>(A) What is the intent<br>(B) What is actually in place		Notes
		(A) The description correctly describes what is intended in the country (intention)<br>**1. Very strongly disagree (no doubts at all)**<br>**2. Strongly disagree (minor doubts)**<br>**3. Disagree (some doubts, but still largely inaccurate)**<br>**4. Agree (some doubts, but still largely accurate)**<br>**5. Strongly agree (minor doubts)**<br>**6. Very strongly agree (no doubts at all)**	***(B) The description correctly describes the situation in practice (actuality)***<br>**1. Very strongly disagree (no doubts at all)**<br>**2. Strongly disagree (minor doubts)**<br>**3. Disagree (some doubts, but still largely inaccurate)**<br>**4. Agree (some doubts, but still largely accurate)**<br>**5. Strongly agree (minor doubts)**<br>**6. Very strongly agree (no doubts at all)**	
PHC workforce	There are adequately trained and motivated workforce across the 13 core cadres to provide both primary care, and hospital-based 1st point of care services. The 13 cadres include medical doctors, nurses/midwives, dental, pharmaceutical, medical assistants, laboratory, imaging technicians, physiotherapy, optical, paramedics, nutritionists, environmentalists and community workers			

Dimension	Description of the attributes	Country situation (based on actions carried out in the past 2 years)<br>Defined by 2 parameters:<br>**(A) What is the intent**<br>**(B) What is actually in place**		Notes
		(A) The description correctly describes what is intended in the country (intention)<br>**1. Very strongly disagree (no doubts at all)**<br>**2. Strongly disagree (minor doubts)**<br>**3. Disagree (some doubts, but still largely inaccurate)**<br>**4. Agree (some doubts, but still largely accurate)**<br>**5. Strongly agree (minor doubts)**<br>**6. Very strongly agree (no doubts at all)**	***(B) The description correctly describes the situation in practice (actuality)***<br>**1. Very strongly disagree (no doubts at all)**<br>**2. Strongly disagree (minor doubts)**<br>**3. Disagree (some doubts, but still largely inaccurate)**<br>**4. Agree (some doubts, but still largely accurate)**<br>**5. Strongly agree (minor doubts)**<br>**6. Very strongly agree (no doubts at all)**	
Physical infrastructure	There exists adequate and functional health infrastructure at the primary care and hospital levels, to provide the needed essential services. Infrastructure includes physical buildings, transport, and equipment			
Medicines & other products	There is the required and adequate health products needed to deliver the essential health services needed. Health products include medicines, vaccines, traditional products, medical products of human origin.			
Engagement with private sector	The private sector partners are actively involved in the planning, provision and monitoring of essential services provision.			

Dimension	Description of the attributes	Country situation (based on actions carried out in the past 2 years)<br>Defined by 2 parameters:<br>(A) What is the intent<br>(B) What is actually in place		Notes
		(A) The description correctly describes what is intended in the country (intention)<br>**1. Very strongly disagree (no doubts at all)**<br>**2. Strongly disagree (minor doubts)**<br>**3. Disagree (some doubts, but still largely inaccurate)**<br>**4. Agree (some doubts, but still largely accurate)**<br>**5. Strongly agree (minor doubts)**<br>**6. Very strongly agree (no doubts at all)**	***(B) The description correctly describes the situation in practice (actuality)***<br>**1. Very strongly disagree (no doubts at all)**<br>**2. Strongly disagree (minor doubts)**<br>**3. Disagree (some doubts, but still largely inaccurate)**<br>**4. Agree (some doubts, but still largely accurate)**<br>**5. Strongly agree (minor doubts)**<br>**6. Very strongly agree (no doubts at all)**	
Purchasing and payment systems	The health sector is actively mobilizing public resources, efficiently pooling these and effectively purchasing the needed essential services.			
Digital technologies for health	The health sector is using digital solutions across the full range of health service provision. This includes *Management*: workforce, equipment, operations, health products, information, finances<br>*Services*: Outpatients, diagnosis, imaging, pharmacy, theater, management, clinics			

<table>
<tr><th rowspan="2">Dimension</th><th rowspan="2">Description of the attributes</th><th colspan="2">Country situation (based on actions carried out in the past 2 years)
Defined by 2 parameters:
(A) What is the intent
(B) What is actually in place</th><th>Notes</th></tr>
<tr><th>(A) The description correctly describes what is intended in the country (intention)
1. Very strongly disagree (no doubts at all)
2. Strongly disagree (minor doubts)
3. Disagree (some doubts, but still largely inaccurate)
4. Agree (some doubts, but still largely accurate)
5. Strongly agree (minor doubts)
6. Very strongly agree (no doubts at all)</th><th>(B) The description correctly describes the situation in practice (actuality)
1. Very strongly disagree (no doubts at all)
2. Strongly disagree (minor doubts)
3. Disagree (some doubts, but still largely inaccurate)
4. Agree (some doubts, but still largely accurate)
5. Strongly agree (minor doubts)
6. Very strongly agree (no doubts at all)</th><th></th></tr>
<tr><td>Systems for improving quality of care</td><td>The sector is practicing interventions to improve the perceptions of care, infection prevention, adherence to care standards, and health outcomes</td><td></td><td></td><td></td></tr>
<tr><td>PHC-oriented research</td><td>The sector is carrying out biomedical, and operations research to generate evidence and using this to inform decision making</td><td></td><td></td><td></td></tr>
<tr><td>Monitoring and evaluation</td><td>The sector is conducting regular operational (monthly, quarterly, annual), strategic and policy monitoring and review processes for constant improvement in implementation of essential service interventions</td><td></td><td></td><td></td></tr>
</table>

HEALTH SYSTEM QUALITATIVE INFORMATION NEEDS FOR THE STATE OF PHC REPORT

	**National level**<br>1. Very strongly disagree (no doubts at all)<br>2. Strongly disagree (minor doubts)<br>3. Disagree (some doubts, but still largely inaccurate)<br>4. Agree (some doubts, but still largely accurate)<br>5. Strongly agree (minor doubts)	**Sub-national level (district/county/state/province)**<br>1. Very strongly disagree (no doubts at all)<br>2. Strongly disagree (minor doubts)<br>3. Disagree (some doubts, but still largely inaccurate)<br>4. Agree (some doubts, but still largely accurate)<br>5. Strongly agree (minor doubts)
**GOVERNANCE**<br>This encompasses the oversight and coordination of the health agenda		
Decision space: The health leadership has the authority to mobilize, plan, and deploy staff, infrastructure, health products, and funds based on needs		
Organizational structure: The existing structure is aligned with the delivery of Universal Health Coverage, Health Security, and Health results across the determinants		
Policy, strategic & operational planning: There is a clear policy (over 5 years), strategic (3-5 years) and operational (1 year) planning process used by both Government and partners		
Technical accountability: There is a process that the Government and partners use to hold the health sector stakeholders accountable based on achieving planned deliverables		

	National level<br>1. Very strongly disagree (no doubts at all)<br>2. Strongly disagree (minor doubts)<br>3. Disagree (some doubts, but still largely inaccurate)<br>4. Agree (some doubts, but still largely accurate)<br>5. Strongly agree (minor doubts)	Sub-national level (district/county/state/province)<br>1. Very strongly disagree (no doubts at all)<br>2. Strongly disagree (minor doubts)<br>3. Disagree (some doubts, but still largely inaccurate)<br>4. Agree (some doubts, but still largely accurate)<br>5. Strongly agree (minor doubts)
Social accountability: There is a process individuals and families can use to hold the health sector accountable, based on their ability to deliver on expected needs		
Regulatory framework: The health sector laws, regulations, and other legal instruments are appropriate for facilitating the achievement of Universal Health Coverage, Health Security, and Health results across the determinants		
Stakeholder engagement: The health donors, technical partners, private sector, and civil society partners can engage with and provide inputs to the planning, implementation, and monitoring of the health agenda		
Integrity & public confidence: There are processes in place to build the public trust and respect in the sector		
**HEALTH WORKFORCE**<br>These include medical doctors, nursing/midwifery professionals, dentists & dental assistants, pharmacists & pharmacy technicians/assistants, medical/clinical assistants, laboratory technicians/technologists, imaging technicians, physiotherapists & physiotherapy technicians/assistants, paramedics, and community health workers		
Regulation: There are required regulatory mechanisms to coordinate and manage the production, recruitment, deployment and use of the health workforce		

	**National level**<br>1. **Very strongly disagree (no doubts at all)**<br>2. **Strongly disagree (minor doubts)**<br>3. **Disagree (some doubts, but still largely inaccurate)**<br>4. **Agree (some doubts, but still largely accurate)**<br>5. **Strongly agree (minor doubts)**	**Sub-national level (district/county/state/province)**<br>1. **Very strongly disagree (no doubts at all)**<br>2. **Strongly disagree (minor doubts)**<br>3. **Disagree (some doubts, but still largely inaccurate)**<br>4. **Agree (some doubts, but still largely accurate)**<br>5. **Strongly agree (minor doubts)**
Production: There is the ability to produce the needed health workers		
Norms: There are aspirational goals regarding the different health workers for the country		
Projection: There are health worker targets for the short (1 year) medium (3-5 years) and long (>5 years) term		
Recruitment: There are adequate resources to recruit all the needed health workers who are available on the market		
Deployment: The health leadership has the responsibility to deploy health workers based on need		
Motivation: There are processes to ensure motivation and productivity of the health workers		
Exit: The health sector is able to efficiently remove health workers who are not productive or effective		
**HEALTH INFRASTRUCTURE**<br>These include the physical infrastructure like buildings, equipment, transport infrastructure, ICT infrastructure		
Regulation: There are required regulatory mechanisms to coordinate and manage the production, recruitment, deployment, and use of the health infrastructure		
Production: There is the ability to produce the needed health infrastructure		

	**National level**<br>1. **Very strongly disagree (no doubts at all)**<br>2. **Strongly disagree (minor doubts)**<br>3. **Disagree (some doubts, but still largely inaccurate)**<br>4. **Agree (some doubts, but still largely accurate)**<br>5. **Strongly agree (minor doubts)**	**Sub-national level (district/county/state/province)**<br>1. **Very strongly disagree (no doubts at all)**<br>2. **Strongly disagree (minor doubts)**<br>3. **Disagree (some doubts, but still largely inaccurate)**<br>4. **Agree (some doubts, but still largely accurate)**<br>5. **Strongly agree (minor doubts)**
Norms: There are aspirational goals regarding the different health infrastructure for the country		
Projection: There are health infrastructure targets for the short (1 year) medium (3-5 years) and long (>5 years) term		
Onboarding: There are adequate resources to purchase/build the needed infrastructure		
Deployment: The health leadership has the responsibility to deploy health infrastructure based on need		
Use: There are processes to monitor and ensure functionality and servicing of health infrastructure		
Offboarding: The health sector is able to efficiently get rid of health infrastructure that is no longer productive or effective		
**HEALTH PRODUCTS**<br>These include medicines, vaccines, medical products of human origin, traditional products, medical devices		
Regulation: There are required regulatory mechanisms to coordinate and manage the production, recruitment, deployment and use of the health products		
Production: There is the ability to produce the needed health products		
Norms: There are aspirational goals regarding the different health oroducts for the country		

	**National level**<br>**1. Very strongly disagree (no doubts at all)**<br>**2. Strongly disagree (minor doubts)**<br>**3. Disagree (some doubts, but still largely inaccurate)**<br>**4. Agree (some doubts, but still largely accurate)**<br>**5. Strongly agree (minor doubts)**	**Sub-national level (district/county/state/province)**<br>**1. Very strongly disagree (no doubts at all)**<br>**2. Strongly disagree (minor doubts)**<br>**3. Disagree (some doubts, but still largely inaccurate)**<br>**4. Agree (some doubts, but still largely accurate)**<br>**5. Strongly agree (minor doubts)**
Projection: There are health products targets for the short (1 year) medium (3-5 years) and long (>5 years) term		
Procurement: There are adequate resources to purchase the needed health products		
Distribution: The health leadership has the responsibility and capacity to distribute health products based on need		
Rational use: There are processes to ensure availability, efficacy, effective management and use of health products		
Disposal: There are processes to facilitate the safe, effective and efficient disposal of health products		
**HEALTH INFORMATION**<br>This includes information from the routine facility, civil registration, vital statistics, population surveys, health facility surveys, research		
Generation: There is capacity to produce the needed health data and statistics		
Analysis: There exists the capacity to interpret the generated data/statistics, to explain the emerging trends, and distribution information		
Evidence use: There is the production and use of evidence from health data/statistics to guide decision-making in health		
**HEALTH FINANCING**		

	**National level**<br>**1. Very strongly disagree (no doubts at all)**<br>**2. Strongly disagree (minor doubts)**<br>**3. Disagree (some doubts, but still largely inaccurate)**<br>**4. Agree (some doubts, but still largely accurate)**<br>**5. Strongly agree (minor doubts)**	**Sub-national level (district/county/state/province)**<br>**1. Very strongly disagree (no doubts at all)**<br>**2. Strongly disagree (minor doubts)**<br>**3. Disagree (some doubts, but still largely inaccurate)**<br>**4. Agree (some doubts, but still largely accurate)**<br>**5. Strongly agree (minor doubts)**
This includes Government, donor, private, and out-of-pocket sources		
Mobilization: There are processes to maximize the efficiency and equity of different financing sources		
Pooling: The country has institutional means to bring resources together, to ensure use based on demand while contribution is based on capacity (taxation, Insurance, etc)		
Purchasing: There are processes to link health financing to specified health results (move from input to output/outcome purchasing)		
**SERVICE DELIVERY**<br>This includes systems at the primary care, hospital, management units		
Essential Packages: There is a defined set of essential health interventions based on the needs of the population		
Client-centredness: Health service provision is organized around the needs of the beneficiaries of services		
Infection Prevention: There are processes in all facilities to avoid harm to beneficiaries during the process of care		
Clinical governance: There are therapeutic committees in all facilities to monitor and ensure adherence to care standards		

	National level<br>1. Very strongly disagree (no doubts at all)<br>2. Strongly disagree (minor doubts)<br>3. Disagree (some doubts, but still largely inaccurate)<br>4. Agree (some doubts, but still largely accurate)<br>5. Strongly agree (minor doubts)	Sub-national level (district/county/state/province)<br>1. Very strongly disagree (no doubts at all)<br>2. Strongly disagree (minor doubts)<br>3. Disagree (some doubts, but still largely inaccurate)<br>4. Agree (some doubts, but still largely accurate)<br>5. Strongly agree (minor doubts)
Treatment guidelines: There are standards to care provision and management in use for all health service providers including the private sector		
Licensure: There is a process to assess suitability of facilities to provide services		
Accreditation: There is a stepwise process to facilitate progressive quality improvement towards defined standards		
Supervision: There is a process to support, mentor and encourage continuous improvement in service provision capacity		
**HEALTH SYSTEMS CAPACITY**		
Access: There are adequate targeted interventions to address access barriers including physical, financial, and socio-cultural.		
Demand There are adequate targeted interventions to generate demand from the population, focused on increased health-seeking behavior and individual health action		
Quality: There are adequate targeted interventions to enhance the quality of care; especially related to user experience, user safety, and effectiveness of care		
Resilience: There are adequate targeted interventions that foster resilience, both targeted resilience (aimed at known shock events) and		

	**National level**<br>**1. Very strongly disagree (no doubts at all)**<br>**2. Strongly disagree (minor doubts)**<br>**3. Disagree (some doubts, but still largely inaccurate)**<br>**4. Agree (some doubts, but still largely accurate)**<br>**5. Strongly agree (minor doubts)**	**Sub-national level (district/county/state/province)**<br>**1. Very strongly disagree (no doubts at all)**<br>**2. Strongly disagree (minor doubts)**<br>**3. Disagree (some doubts, but still largely inaccurate)**<br>**4. Agree (some doubts, but still largely accurate)**<br>**5. Strongly agree (minor doubts)**
inherent resilience (capacity to handle, adapt and transform when facing unpredictable shock events)		

Appendix 2: List of participants by country and affiliation

Country	Government	Partners	Total
Algeria		1	1
Angola	3	2	5
Benin	1	1	2
Botswana	6	1	7
Burundi	2	2	4
Cabo Verde	5	1	6
Cameroon	5	1	6
Central African Republic	1	3	4
Chad		1	1
Comoros	4	2	6
Ivory Coast	2	2	4
Democratic Republic of Congo		3	3
Equatorial Guinea	3	2	6
Eritrea	1		1
Eswatini	3	2	5
Ethiopia	3	1	4
Gabon	1	2	3
Gambia	4	1	5
Ghana	1	2	3
Guinea	1	1	2
Guinea Bissau	1	3	1
Kenya	1	3	1
Lesotho	1	3	3
Liberia		1	1
Madagascar	3	3	6
Malawi	2		2
Mali		2	2
Mauritania	2	1	3
Mauritius	4	2	6
Mozambique	3	3	6
Namibia	3	3	6
Niger	1		1
Nigeria		3	3
Rwanda	1	3	4
Sao Tome And Principe	1		1
Senegal	7	2	9

Seychelles	1	1	2
Sierra Leone	1	2	3
South Africa	1		1
South Sudan	1	1	2
Togo	1	1	2
Uganda	1		1
Zambia	3	2	5
Zimbabwe	2	2	4
		**Total**	**153**

Appendix 3: The constructs and their variables

Sno	Construct	variable name	Variables
1	PHC component	phc 1	Multsectoriality
		phc 2	Empowerement
		phc 3	Integration
2	Strategic levers	Strat 1	political commitment leadership practise
		Strat 2	governance policy frameworks practise
		Strat 3	funding and allocation of resources practise
		Strat 4	engagement of communities practise
3	Health investments	Invest 1	Governance
		Invest 2	Human Resources for Health
		Invest 3	Infrastructure
		Invest 4	Health products
		Invest 5	Health Information Systems
		Invest 6	Health Financing and Insurance
		Invest 7	Service Delivery Systems
4	Operational levers	Opr 1	models of care practise
		Opr 2	phc workforce practise
		Opr 3	physical infrastructure practise
		Opr 4	medicines other products practice
		Opr 5	engagement with private sector practise
		Opr 6	purchasing and payment systems practise
		Opr 7	digital technologies practise
		Opr 8	systems for improving QoC practise
		Opr 9	phc oriented research practise
		Opr 10	monitoring and evaluation practise
5	Functionality	Funct 1	Demand

		Funct 2	Quality
		Funct 3	Access
		Funct 4	Resilience
6	Results	Result 1	Health Security
		Result 2	UHC Service Coverage Index
		Result 3	Determiniants of health
7	multisectorality	Multi_1	multisectorality_geographical_area
		Multi_2	multisectorality_services_covered
		Multi_3	multisectorality_age_cohorts_covered
		Multi_4	multisectorality_vulnerable_pop_covered
8	empowerment	Empower_1	empowerment_geographical_area
		Empower_2	empowerment_services_covered
		Empower_3	empowerment_age_cohorts_covered
		Empower_4	empowerment_vulnerable_pop_covered
9	integration	Intergration_1	integration_geographical_area_covered
		Intergration_2	integration_services_covered
		Intergration_3	integration_age_cohorts_covered
		Intergration_4	integration_vulnerable_pop_covered
10	Governance	Gov_1	Gov_integrity_public_confidence_national
		Gov_2	Gov_social_accountability_national
		Gov_3	Gov_technical_accountability_national
		Gov_4	Gov_organizational_structure_national
		Gov_5	Gov_decision_space_national
		Gov_6	Gov_policy_strategic_operational_planning_national
		Gov_7	Gov_regulatory_framework_national
		Gov_8	Gov_stakeholder_engagement_national
11	Human resources for Health	HRH_1	HRH_motivation_national
		HRH_2	HRH_regulation_national
		HRH_3	HRH_recruitment_national_level

		HRH_4	HRH_norm_national_level
		HRH_5	HRH_projection_national_level
		HRH_6	HRH_deployment_national_level
		HRH_7	HRH_exit_national_level
		HRH_8	HRH_production_national_level
12	Infrastrature	Infrast_1	Infrastructure_use_national_level
		Infrast_2	Infrastructure_deployment_national_level
		Infrast_3	Infrastructure_production_national_level
		Infrast_4	Infrastructure_projection_national_level
		Infrast_5	Infrastructure_norms_national_level
		Infrast_6	Infrastructure_regulation_national_level
		Infrast_7	Infrastructure_onboarding_national_level
		Infrast_8	Infrastructure_offboarding_national_level
13	Health products	Health_p1	Health_products_distribution_national_level
		Health_p2	Health_products_rational_use_national_level
		Health_p3	Health_products_norms_national_level
		Health_p4	Health_products_regulation_national_level
		Health_p5	Health_products_production_national_level
		Health_p6	Health_products_disposal_national_level
		Health_p7	Health_products_projection_national_level
		Health_p8	Health_products_procurement_national_level
14	Health information systems	HIS_1	HIS_generation_national_level
		HIS_2	HIS_evidence_use_national_level
		HIS_3	HIS_analysis_national_level
		HFI_1	HFI_pooling_national_level
		HFI_2	HFI_purchasing_national_level
		HFI_3	HFI_mobilization_national_level
		SDS_1	SDS_IPC_national_level
15	Service delivery systems	SDS_2	SDS_treatment_guidelines_national_level

		SDS_3	SDS_client_centredness_national_level
		SDS_4	SDS_licensure_national_level
		SDS_5	SDS_supervision_national_level
		SDS_6	SDS_accreditation_national_level
		SDS_7	SDS_ehcp_defined_national_level
		SDS_8	SDS_clinical_governance_national_level

Appendix 4: Spearman's rank correlation between the primary data and the related secondary data

Variable	Statistic	UHC SCI	HALE	SPAR	gghed che	gghed gdp	gghed gge	SDG 3c occupations density per 10000	Total HWF density	Nurses and midwives per 10000	Patient safety index score	Quality of care	User experience score	Health Security Index	Political index	Prop. below poverty line (%) -- Inverse	Stunting for under 5 - inverse	Social index
PHC comp	Corr	.213	.212	.232	.141	.168	.167	.090	.113	.149	.142	.261	.194	.298*	.125	.214	.121	.258
	p-value	.155	.157	.120	.351	.264	.266	.554	.456	.323	.348	.080	.197	.045	.408	.153	.424	.084
PHC Comp geo area	Corr	.240	.219	.295*	.096	.149	.134	.045	.042	.098	.082	.268	.250	.313*	.151	.159	.091	.327*
	p-value	.108	.145	.047	.527	.322	.373	.767	.780	.517	.590	.072	.093	.034	.315	.291	.549	.027
PHC Comp Services	Corr	.269	.252	.288	.130	.186	.194	.055	.075	.090	.125	.291*	.216	.400**	.215	.241	.139	.317*
	p-value	.071	.091	.052	.390	.216	.197	.719	.620	.554	.408	.049	.149	.006	.151	.107	.356	.032
PHC age cohort	Corr	.284	.238	.265	.260	.231	.236	.222	.232	.287	.245	.334*	.246	.362*	.058	.323*	.189	.328*
	p-value	.056	.111	.075	.081	.122	.115	.138	.122	.053	.101	.023	.099	.014	.700	.029	.209	.026
PHC vulnerable pop	Corr	.090	.028	.088	.043	.059	.025	.035	.024	.100	.078	.157	.156	.081	.101	.105	.082	.124
	p-value	.551	.853	.559	.778	.697	.870	.816	.873	.509	.608	.296	.299	.592	.506	.489	.587	.412
Multsectoriality index	Corr	.286	.202	.201	.218	.221	.226	.179	.165	.232	.216	.341*	.273	.305*	.070	.279	.170	.282
	p-value	.054	.179	.181	.145	.139	.130	.235	.272	.120	.149	.020	.067	.039	.642	.061	.258	.058
Empowerment index	Corr	.177	.161	.182	.132	.204	.187	.057	.081	.092	.083	.236	.166	.261	.112	.183	.074	.275
	p-value	.240	.284	.225	.383	.173	.213	.709	.593	.543	.581	.114	.270	.080	.459	.225	.624	.065
Integration index	Corr	.155	.140	.223	.010	.002	.010	.011	.022	.083	.078	.096	.039	.192	.172	.080	.053	.175
	p-value	.305	.354	.136	.946	.992	.946	.942	.887	.584	.605	.524	.798	.200	.254	.599	.725	.245
St levers intention	Corr	.146	.123	.320*	.041	-.101	-.004	.124	.131	.214	-.012	.048	-.040	.261	.059	.157	.007	.167
	p-value	.333	.417	.030	.786	.505	.981	.410	.385	.152	.938	.752	.791	.080	.696	.296	.965	.267
St levers practise	Corr	.365*	.391**	.445**	.187	.184	.169	.240	.226	.322*	.165	.404**	.186	.520**	.025	.290	.148	.348*
	p-value	.013	.007	.002	.213	.222	.262	.108	.131	.029	.273	.005	.217	.000	.869	.050	.328	.018

Variable	Statistic	UHC SCI	HALE	SPAR	gghed che	gghed gdp	gghed gge	SDG 3c occupations density per 10000	Total HWF density	Nurses and midwives per 10000	Patient safety index score	Quality of care	User experience score	Health Security Index	Political index	Prop. below poverty line (%) -- Inverse	Stunting for under 5 - inverse	Social index
Op levers intention	Corr	.159	.102	.400**	.143	.060	.106	.088	.052	.192	.067	.220	.190	.237	-.074	.152	-.176	.145
	p-value	.291	.501	.006	.343	.691	.483	.562	.731	.201	.659	.142	.205	.113	.623	.313	.243	.338
Op levers practise	Corr	.292*	.384**	.470**	.367*	.313*	.305*	.312*	.251	.369*	.096	.329*	.279	.524**	-.032	.413**	.012	.358*
	p-value	.049	.008	.001	.012	.034	.039	.035	.092	.012	.528	.026	.060	.000	.835	.004	.938	.015
Gov National	Corr	.241	.324*	.314*	.141	.064	.067	.148	.152	.198	.017	.242	.093	.391**	-.211	.256	.055	.315*
	p-value	.107	.028	.034	.351	.675	.656	.325	.314	.187	.911	.105	.537	.007	.159	.086	.715	.033
Gov sub national	Corr	.269	.436**	.390**	.225	.134	.139	.226	.173	.252	-.017	.274	.107	.526**	-.196	.362*	.065	.311*
	p-value	.071	.002	.007	.133	.375	.358	.132	.249	.091	.911	.065	.478	.000	.193	.013	.668	.035
HRH national	Corr	.244	.352*	.272	.224	.092	.159	.164	.138	.270	.075	.172	.062	.170	-.160	.354*	.123	.365*
	p-value	.102	.017	.067	.135	.544	.292	.277	.360	.070	.621	.254	.682	.259	.288	.016	.415	.013
HRH sub national	Corr	.260	.376*	.455**	.455**	.264	.295*	.250	.238	.346*	.060	.272	.117	.412**	-.157	.321*	.020	.331*
	p-value	.081	.010	.001	.039	.077	.047	.094	.111	.019	.693	.067	.438	.004	.297	.030	.896	.025
Infrastructure national	Corr	.225	.253	.337*	.185	.069	.120	.039	.037	.121	.081	.202	.116	.298*	-.104	.398**	.001	.301*
	p-value	.132	.090	.022	.218	.651	.429	.799	.806	.424	.593	.179	.443	.045	.491	.006	.995	.042
Infrastructure sub national	Corr	.268	.309*	.466**	.307*	.229	.268	.159	.163	.249	-.013	.196	.118	.458**	-.124	.353*	-.005	.336*
	p-value	.071	.037	.001	.038	.125	.072	.290	.279	.095	.932	.192	.436	.001	.412	.016	.972	.022
Health products national	Corr	.141	.088	.216	.146	-.003	.053	.019	.010	.085	.130	.175	.055	.281	-.019	.307*	-.005	.276
	p-value	.350	.562	.149	.333	.987	.727	.899	.946	.577	.390	.246	.718	.058	.900	.038	.973	.063
	Corr	.202	.175	.328*	.256	.177	.209	.142	.114	.215	.088	.200	.105	.441**	.025	.311*	.036	.369*

Variable	Statistic	UHC SCI	HALE	SPAR	gghed che	gghed gdp	gghed gge	SDG 3c occupations density per 10000	Total HWF density	Nurses and midwives per 10000	Patient safety index score	Quality of care	User experience score	Health Security Index	Political index	Prop. below poverty line (%) -- Inverse	Stunting for under 5 - inverse	Social index
Health products sub national	p-value	.178	.246	.026	.086	.239	.163	.348	.452	.152	.561	.182	.487	.002	.867	.036	.813	.012
HIS National	Corr	-.010	.123	.112	-.066	-.179	-.115	-.158	-.132	-.073	-.008	.073	.060	.169	-.192	.122	-.175	.041
	p-value	.949	.416	.459	.661	.234	.447	.295	.382	.632	.960	.628	.691	.263	.201	.419	.245	.784
HIS sub national	Corr	.068	.276	.255	.056	.029	.051	.021	.027	.086	-.108	.156	.190	.366*	-.162	.236	-.169	.157
	p-value	.655	.063	.088	.711	.848	.734	.887	.856	.568	.476	.302	.206	.012	.282	.115	.262	.298
HFI national	Corr	.203	.200	.409**	.252	.215	.222	.121	.186	.186	.058	.220	.006	.421**	-.245	.374*	-.043	.269
	p-value	.177	.182	.005	.091	.152	.138	.424	.215	.216	.700	.142	.970	.004	.101	.011	.777	.070
HFI sub national	Corr	.193	.252	.402**	.332*	.317*	.361*	.197	.230	.261	-.015	.223	.142	.544**	-.093	.401**	-.042	.316*
	p-value	.199	.092	.006	.024	.032	.014	.189	.124	.080	.919	.136	.348	.000	.538	.006	.783	.033
SDS national	Corr	.272	.161	.327*	.236	.150	.164	.228	.232	.251	.153	.272	.057	.467**	-.096	.318*	.004	.315*
	p-value	.067	.286	.026	.115	.320	.276	.128	.121	.092	.310	.068	.708	.001	.525	.031	.980	.033
SDS sub-national	Corr	.284	.208	.410**	.247	.216	.225	.291*	.299*	.291*	.034	.242	.049	.591**	-.049	.321*	-.044	.380**
	p-value	.056	.166	.005	.098	.150	.133	.050	.043	.050	.822	.105	.744	.000	.746	.030	.771	.009
Ser Capacity demand national level	Corr	.319*	.411**	.286	.268	.101	.157	.272	.294*	.360*	.056	.213	-.059	.258	-.299*	.303*	.079	.356*
	p-value	.031	.005	.054	.072	.503	.298	.068	.047	.014	.710	.156	.697	.083	.044	.041	.602	.015
Ser Capacity demand sub national level	Corr	.276	.475**	.390**	.259	.098	.173	.249	.233	.315*	-.122	.138	-.004	.380**	-.172	.367*	.009	.366*
	p-value	.064	.001	.007	.083	.517	.251	.095	.119	.033	.419	.359	.980	.009	.253	.012	.955	.012
Ser capacity quality national level	Corr	.391**	.326*	.243	.373*	.315*	.376*	.140	.215	.189	.245	.420**	.152	.282	-.123	.316*	.051	.329*
	p-value	.007	.027	.104	.011	.033	.010	.352	.151	.209	.100	.004	.313	.057	.417	.032	.738	.026

Variable	Statistic	UHC SCI	HALE	SPAR	gghed che	gghed gdp	gghed gge	SDG 3c occupations density per 10000	Total HWF density	Nurses and midwives per 10000	Patient safety index score	Quality of care	User experience score	Health Security Index	Political index	Prop. below poverty line (%) -- Inverse	Stunting for under 5 - inverse	Social index
Ser capacity quality sub national level	Corr	.421**	.349*	.420**	.453**	.444**	.418**	.373*	.416**	.414**	.151	.478**	.286	.419**	.045	.371*	.087	.412**
	p-value	.004	.017	.004	.002	.002	.004	.011	.004	.004	.317	.001	.054	.004	.767	.011	.567	.004
Ser capacity access national level	Corr	.227	.282	.201	.301*	.223	.227	.103	.118	.134	.053	.254	.147	.207	-.047	.360*	.063	.271
	p-value	.129	.058	.180	.042	.136	.129	.494	.437	.374	.726	.089	.331	.168	.756	.014	.675	.068
Ser capacity access sub national level	Corr	.222	.442**	.393**	.291*	.236	.283	.104	.120	.156	-.012	.269	.095	.356*	-.044	.434**	.006	.323*
	p-value	.139	.002	.007	.050	.114	.057	.492	.427	.302	.937	.070	.531	.015	.773	.003	.967	.028
Ser capacity resilience national level	Corr	.403**	.419**	.255	.269	.267	.231	.187	.244	.245	.301*	.428**	.026	.223	-.005	.278	.193	.276
	p-value	.005	.004	.088	.070	.073	.122	.214	.103	.100	.042	.003	.862	.137	.974	.061	.198	.063
Ser capacity resilience sub national level	Corr	.467**	.375*	.311*	.406**	.432**	.412**	.310*	.344*	.339*	.327*	.446**	.065	.428**	.115	.276	.231	.339*
	p-value	.001	.010	.036	.005	.003	.004	.036	.019	.021	.026	.002	.669	.003	.446	.064	.123	.021
	N	46	46	46	46	46	46	46	46	46	46	46	46	46	46	46	46	46

**. Correlation is significant at the 0.01 level (2-tailed).

*. Correlation is significant at the 0.05 level (2-tailed).

Appendix 5 : Reliability of the model parameter

Model node	alpha	rhoC	AVE	rhoA
PHC components	0.879	0.925	0.804	0.89
Strategic levers	0.79	0.865	0.617	0.799
Investments	0.949	0.958	0.765	0.956
operational levers	0.886	0.908	0.501	0.893
Functionality	0.865	0.908	0.712	0.868
Results	0.718	0.813	0.595	0.856

Note: Alpha, rhoC, and rhoA should exceed 0.7 while AVE should exceed 0.5

Appendix 6: The percentage gap between intention and practice of the strategic levers

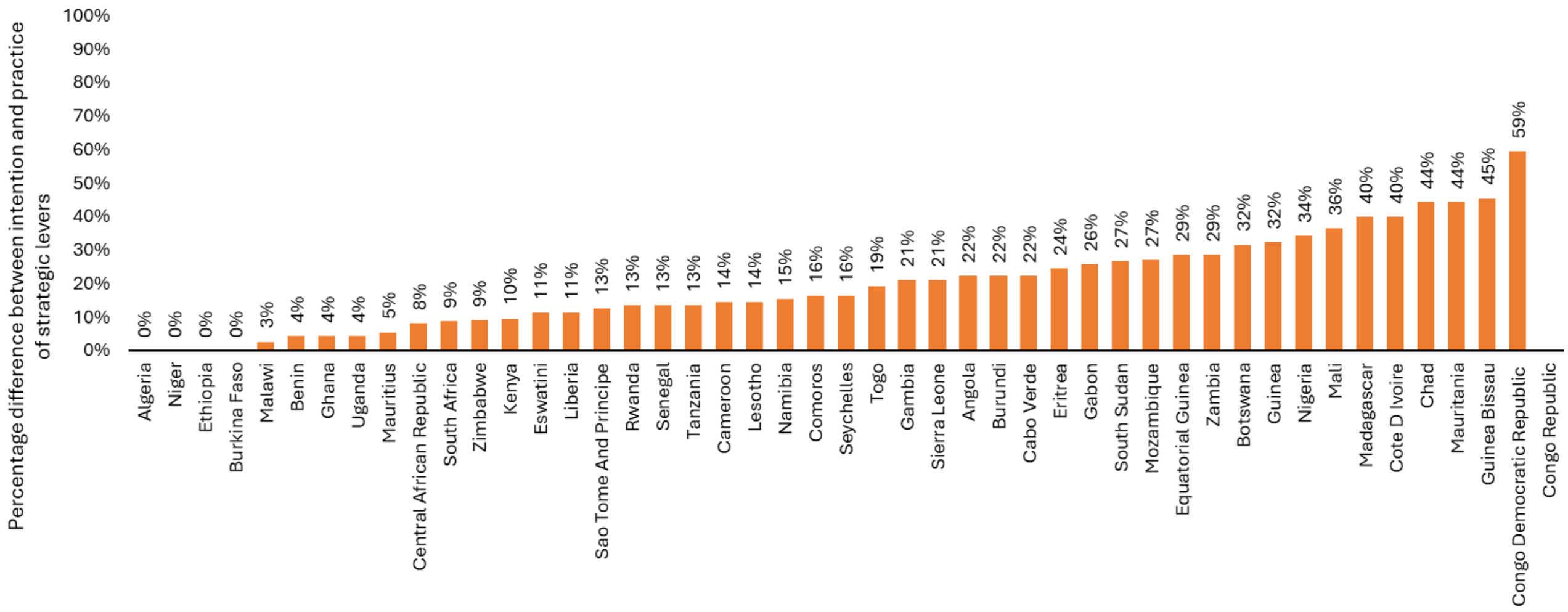

Appendix 7: The percentage gap between intention and practice of the operational levers

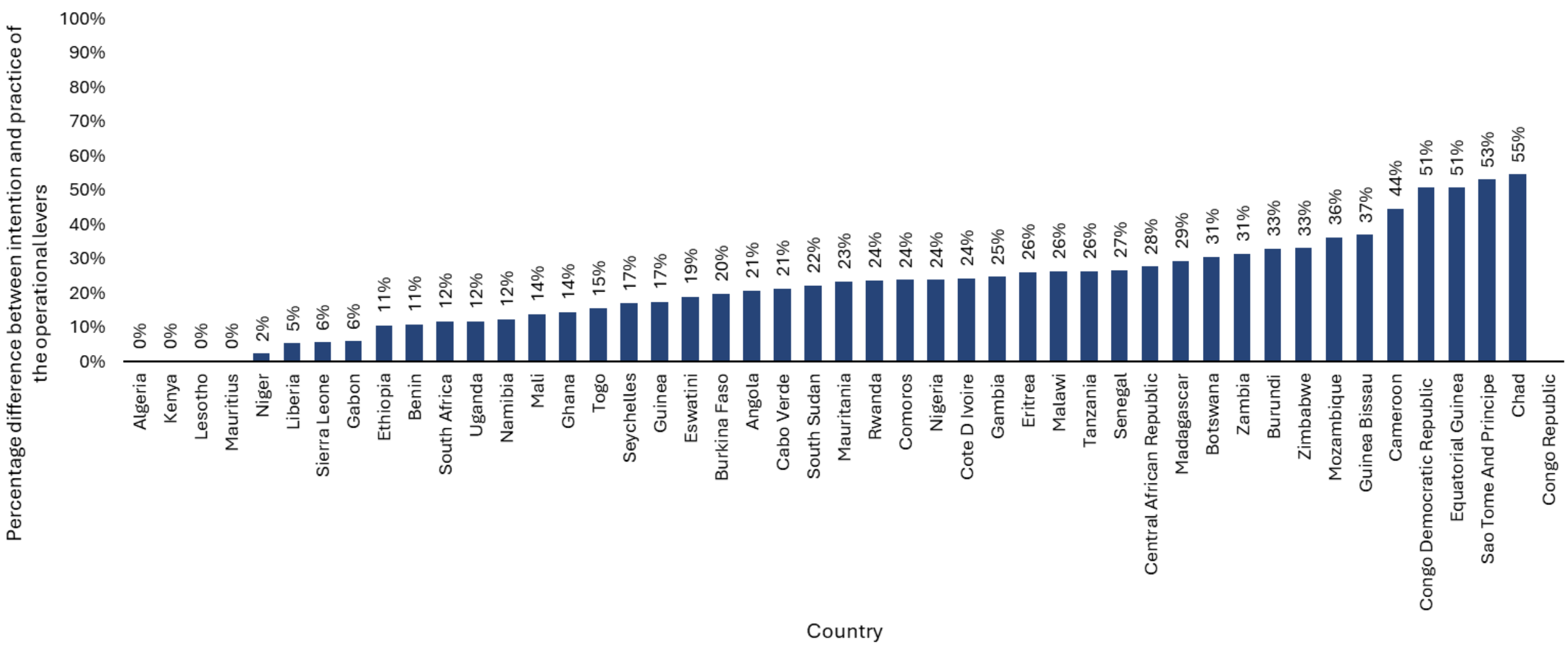

Appendix 8: The intention and practice of strategic levers to PHC

(a) Intension *(b) Practice*

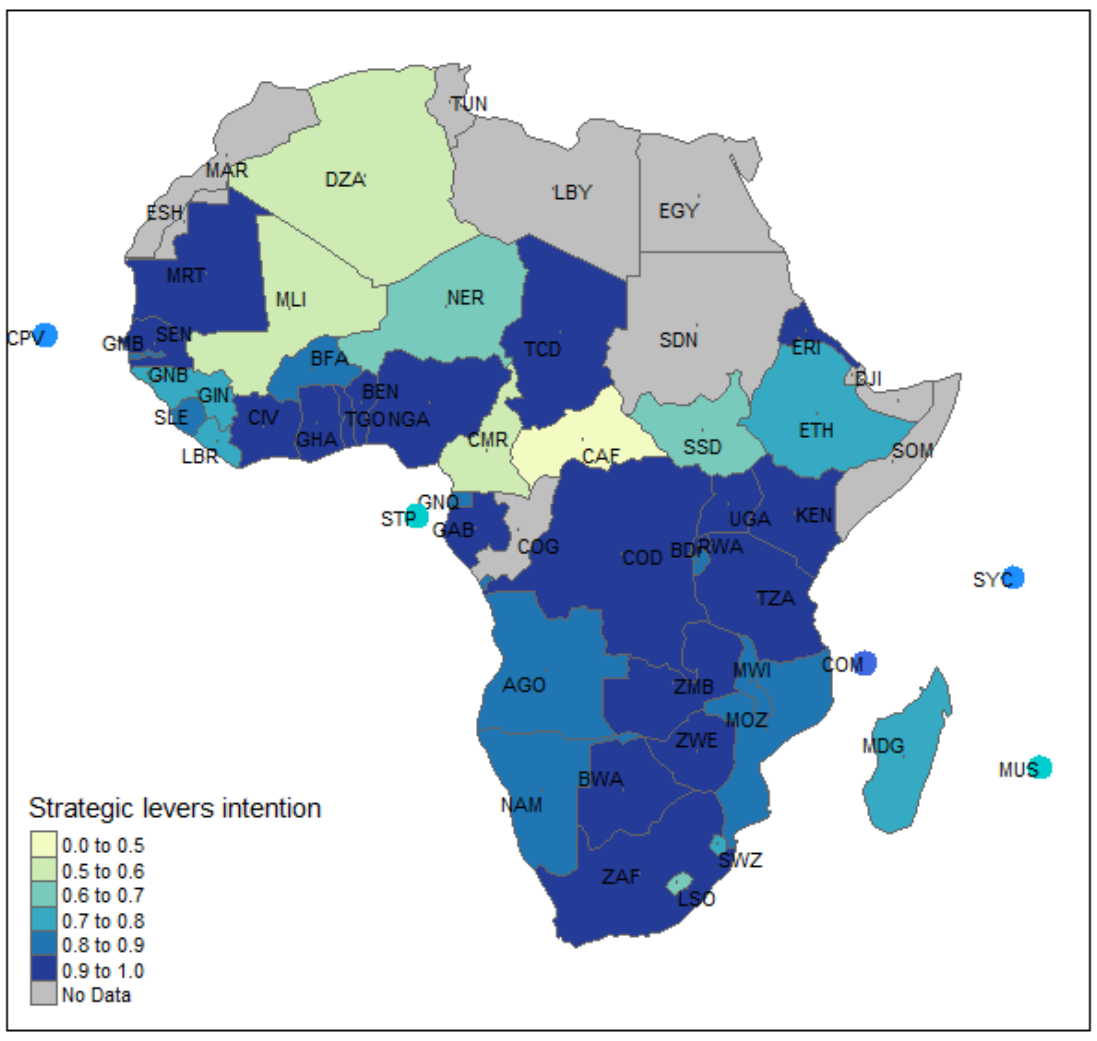

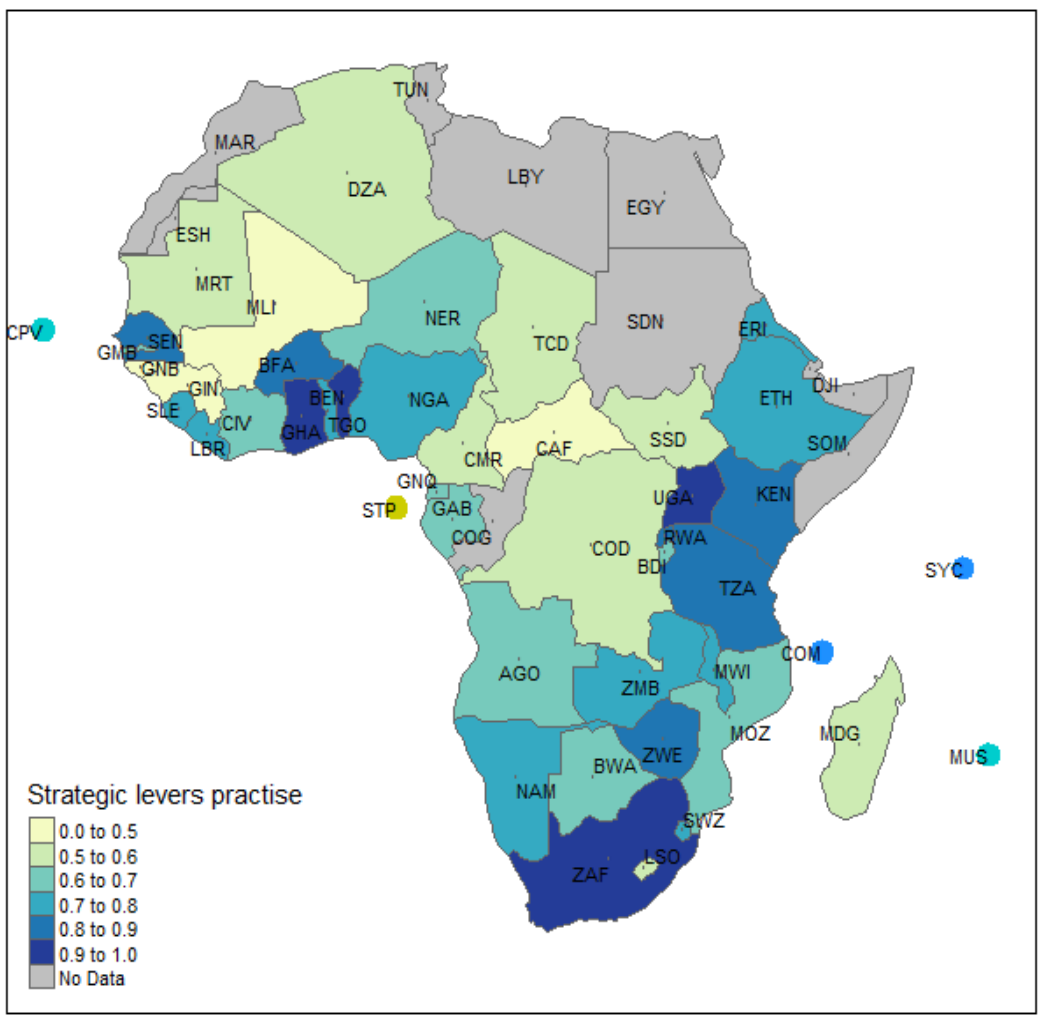

Appendix 9: The intention and practice of operational levers to the PHC approach

(a) Intention *(b) Practice*

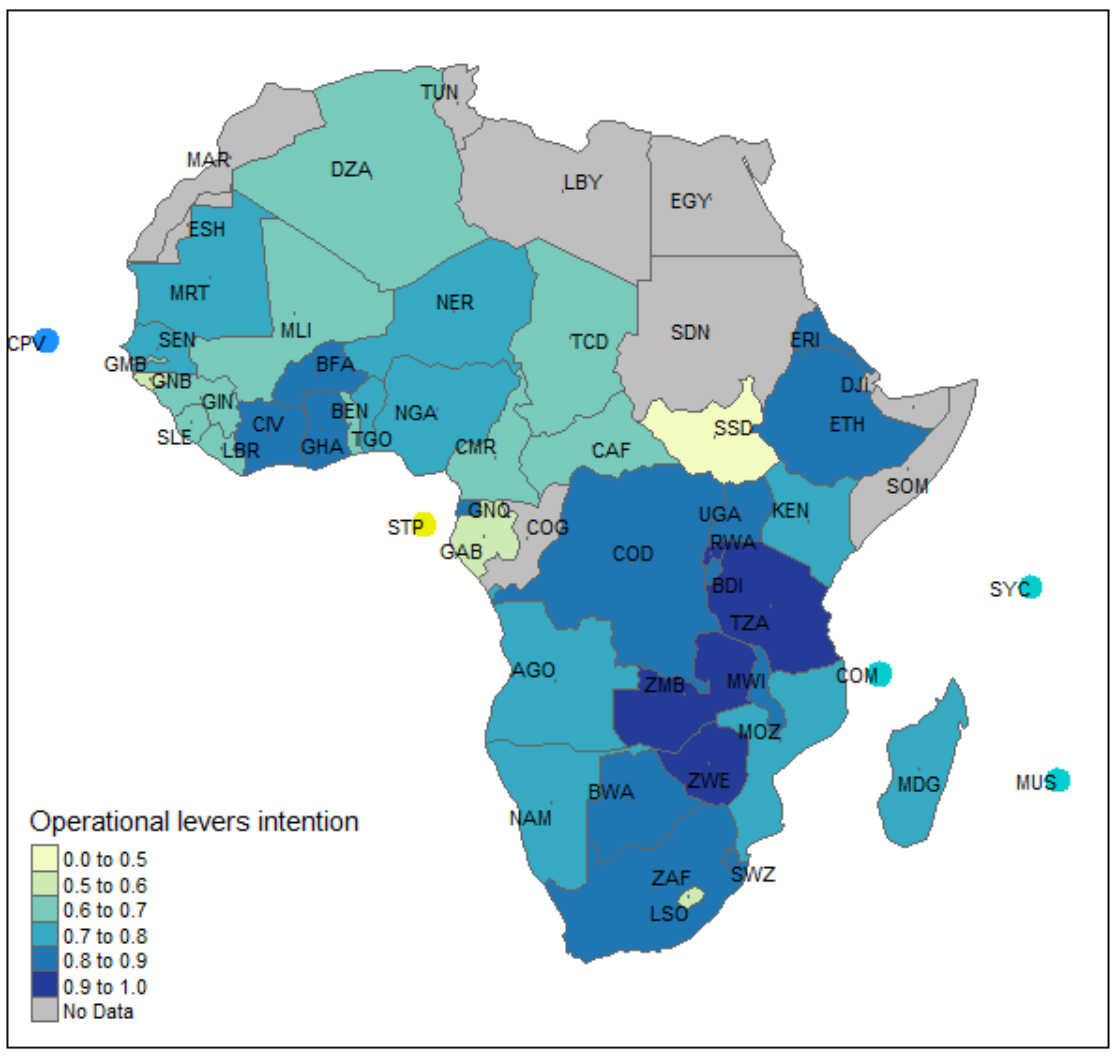

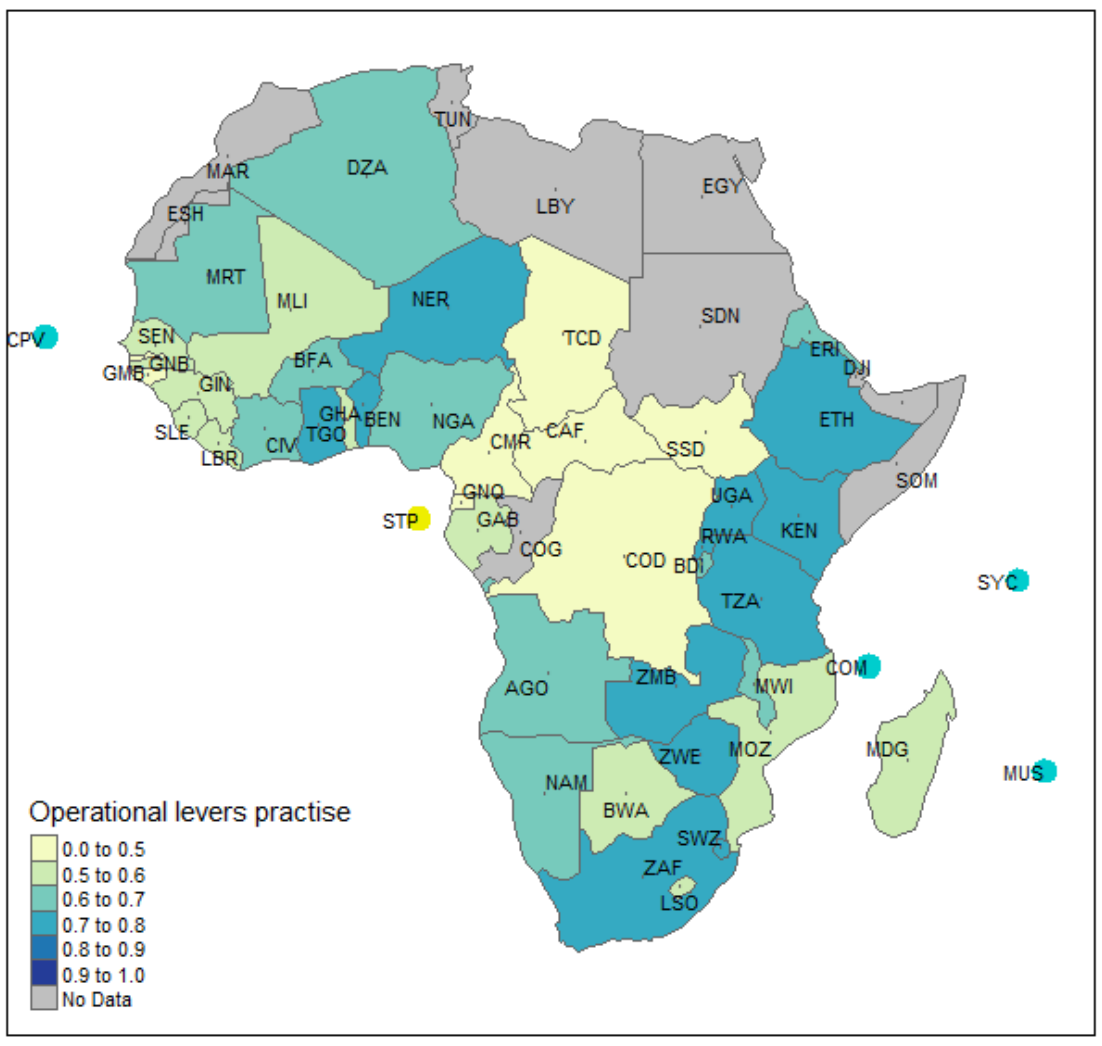

Appendix 10. Classification Criteria for Country Attributes

1. Land Size

I. Large: > 1 million km² (9 countries)

II. Medium: 250,000 – 1 million km² (15 countries)

III. Small: < 250,000 km² (22 countries)

2. Population Density (Tertiles)

I. Low: < 44/km² (15 countries)

II. Medium: 44 – 115/km² (16 countries)

III. High: > 115/km² (15 countries)

3. Income Category

I. High Income: 2 countries

II. Upper-Middle Income: 7 countries

III. Lower-Middle Income: 16 countries

IV. Low Income: 21 countries

4. Land Type

I. Island States: 6 countries

II. Landlocked States: 16 countries

III. Non-Landlocked Coastal States: 24 countries

IV.

Table: Descriptives of Country Attributes

SNo	Country	Land Size	Land Area (km²)	Density Cat	Pop Density (/km²)	Income Level	Income Category	Land Type
1	Algeria	Large	2381741	Low	18.3	Upper-Middle Income	Upper-Middle	Non-Landlocked
2	Angola	Large	1246700	Low	26	Upper-Middle Income	Upper-Middle	Non-Landlocked
3	Benin	Small	114763	High	108.9	Lower-Middle Income	Lower-Middle	Non-Landlocked
4	Botswana	Medium	581730	Low	4.2	Upper-Middle Income	Upper-Middle	Landlocked
5	Burkina Faso	Medium	274000	Medium	76.1	Low Income	Low	Landlocked
6	Burundi	Small	27834	High	477.2	Low Income	Low	Landlocked
7	Cabo Verde	Small	4033	High	138.3	Lower-Middle Income	Lower-Middle	Island
8	Cameroon	Medium	475442	Medium	56	Lower-Middle Income	Lower-Middle	Non-Landlocked
9	Central African Republic	Medium	622984	Low	8	Low Income	Low	Landlocked
10	Chad	Large	1284000	Low	13	Low Income	Low	Landlocked
11	Comoros	Small	1861	High	457	Lower-Middle Income	Lower-Middle	Island
12	Democratic Republic of Congo	Large	2344858	Medium	40	Low Income	Low	Non-Landlocked

SN o	Country	Land Size	Land Area (km²)	Density Cat	Pop Density (/km²)	Income Level	Income Category	Land Type
13	Equatorial Guinea	Small	28051	Medium	50	Upper-Middle Income	Upper-Middle	Non-Landlocked
14	Eritrea	Small	117600	Low	35	Low Income	Low	Non-Landlocked
15	Eswatini	Small	17364	Medium	68.2	Lower-Middle Income	Lower-Middle	Landlocked
16	Ethiopia	Large	1104300	Medium	102.5	Low Income	Low	Landlocked
17	Gabon	Medium	267667	Low	9	Upper-Middle Income	Upper-Middle	Non-Landlocked
18	Gambia	Small	11300	High	239	Low Income	Low	Non-Landlocked
19	Ghana	Small	238533	High	143	Lower-Middle Income	Lower-Middle	Non-Landlocked
20	Guinea	Small	245857	Medium	55.1	Low Income	Low	Non-Landlocked
21	Guinea Bissau	Small	36125	Medium	70	Low Income	Low	Non-Landlocked
22	Ivory Coast	Medium	322463	Medium	83	Lower-Middle Income	Lower-Middle	Non-Landlocked
23	Kenya	Medium	580367	Medium	96	Lower-Middle Income	Lower-Middle	Non-Landlocked
24	Lesotho	Small	30355	Medium	71.2	Lower-Middle Income	Lower-Middle	Landlocked
25	Liberia	Small	111369	Medium	52.1	Low Income	Low	Non-Landlocked
26	Madagascar	Medium	587041	Medium	48	Low Income	Low	Island
27	Malawi	Small	118484	High	203	Low Income	Low	Landlocked
28	Mali	Large	1240192	Low	17	Low Income	Low	Landlocked
29	Mauritania	Large	1030700	Low	4.5	Lower-Middle Income	Lower-Middle	Non-Landlocked
30	Mauritius	Small	2040	High	626.3	High Income	High	Island
31	Mozambique	Medium	799380	Low	38	Low Income	Low	Non-Landlocked
32	Namibia	Medium	825615	Low	3.2	Upper-Middle Income	Upper-Middle	Non-Landlocked
33	Niger	Large	1267000	Low	19.5	Low Income	Low	Landlocked
34	Nigeria	Medium	923768	High	226	Lower-Middle Income	Lower-Middle	Non-Landlocked
35	Rwanda	Small	26338	High	525.2	Low Income	Low	Landlocked
36	Sao Tome and Principe	Small	964	High	228	Lower-Middle Income	Lower-Middle	Island
37	Senegal	Small	196722	Medium	85	Lower-Middle Income	Lower-Middle	Non-Landlocked
38	Seychelles	Small	459	High	210	High Income	High	Island
39	Sierra Leone	Small	72300	High	108.1	Low Income	Low	Non-Landlocked
40	South Africa	Large	1220000	Medium	49.8	Upper-Middle Income	Upper-Middle	Non-Landlocked
41	South Sudan	Medium	644329	Low	18	Low Income	Low	Landlocked
42	Tanzania	Medium	947300	Medium	67.6	Lower-Middle Income	Lower-Middle	Non-Landlocked
43	Togo	Small	56785	High	152	Low Income	Low	Non-Landlocked
44	Uganda	Small	241550	High	229.5	Low Income	Low	Landlocked
45	Zambia	Medium	752612	Low	25	Lower-Middle Income	Lower-Middle	Landlocked
46	Zimbabwe	Medium	390757	Low	38.4	Lower-Middle Income	Lower-Middle	Landlocked

Appendix 11: Impact of individual revitalized PHC approach drivers on investment-functionality-results pathway

Model 2 (a): Impact of PHC components

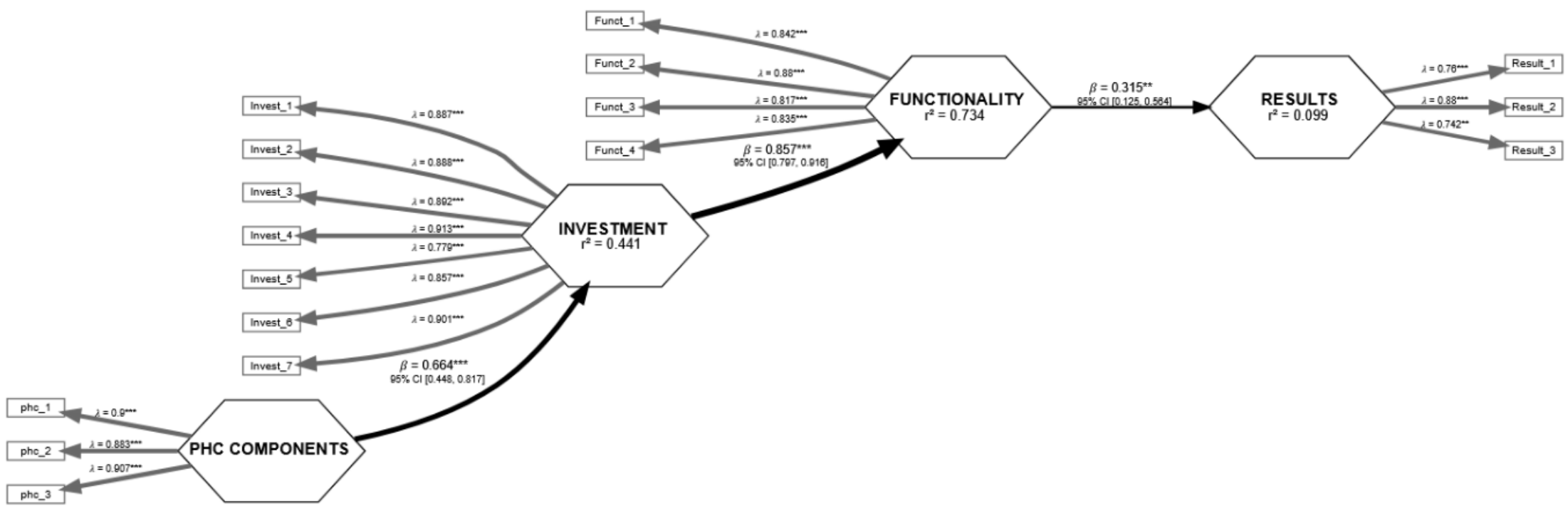

Model 2(b): Impact of strategic levers

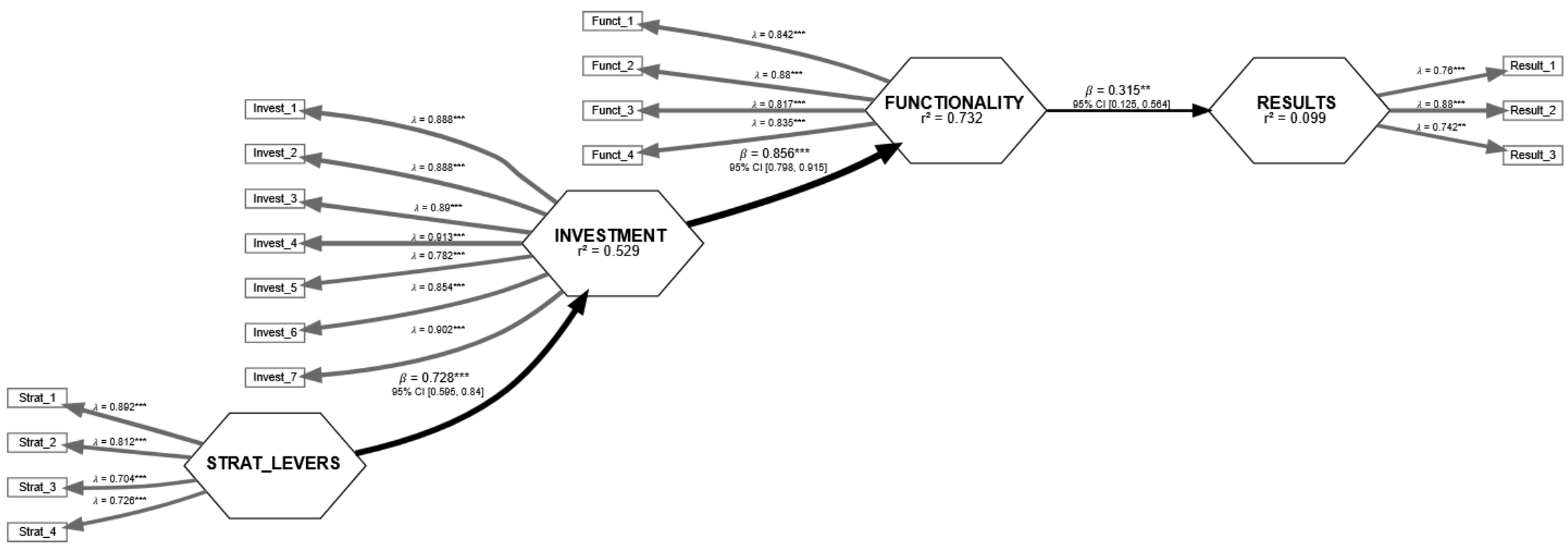

Model 2(c): Impact of operational levers

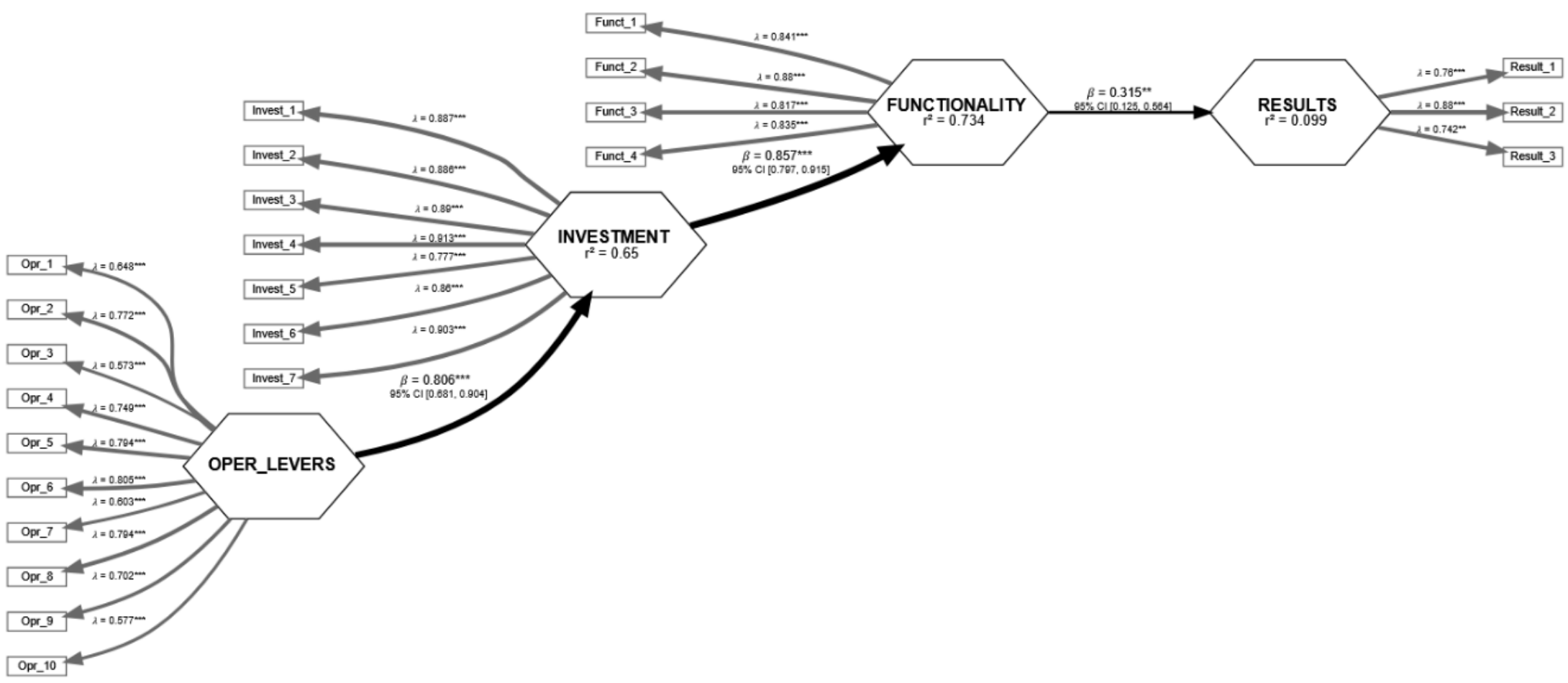

Appendix 12: The key of models 1, 2 and 3

PHC components: phc 1: Multsectoriality, phc 2: Empowerment, phc 3: Integration

Strategic levers: Strat 1: political commitment leadership practise, Strat 2: governance policy frameworks practise, Strat 3: funding and allocation of resources practise, Strat 4: engagement of communities' practise

Investments: Invest 1: Governance, Invest 2: Human Resources for Health, Invest 3: Infrastructure, Invest 4: Health products, Invest 5: Health Information Systems, Invest 6: Health Financing and Insurance, Invest 7: Service Delivery Systems

Operational levers: Opr 1: models of care practise, Opr 2: phc workforce practise, Opr 3: physical infrastructure practise, Opr 4: medicines other products practice, Opr 5: engagement with private sector practise, Opr 6: purchasing and payment systems practise, Opr 7: digital technologies practise, Opr 8: systems for improving QoC practise, Opr 9: phc oriented research practise, Opr 10: monitoring and evaluation practise

Functionality: Funct_1: Demand, Funct_2: Quality, Funct_3: Access, Funct_4: Resilience

Health results: Result 1: Health Security index, Result 2: UHC Service Coverage Index, Result 3: Determinants of health index
